# Genome variation in tick infestation and cryptic divergence in Tunisian indigenous sheep

**DOI:** 10.1186/s12864-022-08321-1

**Published:** 2022-02-28

**Authors:** Abulgasim M. Ahbara, Médiha Khamassi Khbou, Rihab Rhomdhane, Limam Sassi, Mohamed Gharbi, Aynalem Haile, Mourad Rekik, Barbara Rischkowsky, Joram M. Mwacharo

**Affiliations:** 1 Small Ruminant Genomics, International Centre for Agricultural Research in the Dry Areas (ICARDA), Addis Ababa, Ethiopia; 2 Department of Zoology, Faculty of Sciences, Misurata University, Misurata, Libya; 3 Lab. of Infectious Animal Diseases, Zoonosis and Sanitary Regulation, Univ. Manouba, Institution of Agricultural Research and Higher Education, National School of Veterinary Medicine of Sidi Thabet, 2020 Sidi Thabet, Tunisia; 4 Laboratoire de Parasitologie, Institution de La Recherche et de L’Enseignement Supérieur Agricoles, Univ. Manouba, Ecole Nationale de Médecine Vétérinaire de Sidi Thabet, 2020 Sidi Thabet, Tunisia; 5 Animal and Veterinary Sciences Group, SRUC and Centre for Tropical Livestock Genetics and Health (CTLGH), Roslin Institute, Midlothian, Scotland

## Abstract

**Background:**

Ticks are obligate haematophagous ectoparasites considered second to mosquitos as vectors and reservoirs of multiple pathogens of global concern. Individual variation in tick infestation has been reported in indigenous sheep, but its genetic control remains unknown.

**Results:**

Here, we report 397 genome-wide signatures of selection overlapping 991 genes from the analysis, using ROH, LR-GWAS, XP-EHH, and F_ST_, of 600 K SNP genotype data from 165 Tunisian sheep showing high and low levels of tick infestations and piroplasm infections. We consider 45 signatures that are detected by consensus results of at least two methods as high-confidence selection regions. These spanned 104 genes which included immune system function genes, solute carriers and chemokine receptor. One region spanned *STX5*, that has been associated with tick resistance in cattle, implicating it as a prime candidate in sheep. We also observed *RAB6B* and *TF* in a high confidence candidate region that has been associated with growth traits suggesting natural selection is enhancing growth and developmental stability under tick challenge. The analysis also revealed fine-scale genome structure indicative of cryptic divergence in Tunisian sheep.

**Conclusions:**

Our findings provide a genomic reference that can enhance the understanding of the genetic architecture of tick resistance and cryptic divergence in indigenous African sheep.

**Supplementary Information:**

The online version contains supplementary material available at 10.1186/s12864-022-08321-1.

## Background

Ticks are among the most ancient acari and possibly the earliest to evolve blood-feeding capacity through the development of immune-active proteins and lipids [1–5]. These induce vasodilatory, anti-haemostatic and immunomodulatory activities that guarantees their successful engorgement and acquisition of a blood meal [1–5]. Compared to other arthropod vectors, ticks transmit a greater variety of pathogenic microorganisms including protozoa, bacteria and viruses, implicated in severe infections in humans and animals [6, 7]. Globally, ticks and tick-borne infections (T-TBI) are a major health impediment to livestock performance especially in the (sub-)tropics where the host, vector and pathogen overlap. So far, the impact of ticks on sheep production has not been investigated to the same extent as in cattle. The available estimates show that T-TBI affect about 80% of world’s cattle, with a global annual loss approximating US$7 billion [8, 9]. T-TBI negatively impact cattle production by reducing total milk yields by up to 23% [10] and the value of skins and hides by 20-30% [11, 12]. The negative effects of ticks on liveweights are also high. On average, an engorged female tick results in a body weight reduction of 1.37 ± 0.25 and 1.18 ± 0.21 g in *Bos taurus* and *B. taurus* x *B. indicus* crossbred cattle [13], respectively. In China estimates show that the total annual losses due to TBI in small ruminants approximates US$70 million [14]. 

Prophylactic use of acaricides is the most common strategy of controlling and eradicating ticks [15, 16]. The (over) use of acaricides has however imposed selection pressure resulting in the development of acaricide-resistant tick strains, environmental contamination, retention of chemical residues in livestock products and increased costs of existing, and of developing and manufacturing new and more potent, acaricides [16–20]. Anti-tick vaccines are a promising option, but they do not confer protection against multiple tick species [7, 21, 22] which is a common occurrence especially in the (sub-)tropics [23–25]. These factors are driving the search for alternative control strategies such as the use of tick-resistant animal genotypes as the host’s natural resistance to ticks can be exploited as a long-term sustainable control option targeting most tick species.

Genetic variation in resistance to parasites has been demonstrated in many livestock species with investigations on individual variation in resistance to T-TBI being the subject of intense research in cattle (see review by [26]). Most of the studies have shown that an effective immunological response (resistance/tolerance) to T-TBI, is genetically determined with a heritability range of 0.05-0.42 (see [26]). For haemopathogens, the immunological response has been associated with the ability to resist the development of anemia following infection [25, 26], variation in the expression of extracellular matrix metalloproteinase [27], differences in chemokines and their receptors and toll-like receptors [28, 29], variation in genes that limit the supply of blood-meal to ticks and genes that enhance innate and adaptive immune responses [30]. Several genes have been proposed as candidates for tick resistance. Lumican (*LUM*) was identified as a potential biomarker for tick resistance in cattle [30, 31] reported two SNPs in *ELTD1* associated with tick burdens in both dairy and beef cattle, and a haplotype of nine tag SNPs and two others associated with tick counts in dairy cattle. The authors also reported haplotypes spanning *ITGA11* associated with tick burdens. Class I antigens of the bovine major histocompatibility complex have also been associated with tick loads [32, 33] and alleles at the bovine lymphocyte antigen (BOLA-DRB3) have been linked with resistance to *Rhipicephalus* (*Boophilus*) *microplus* infestation [34–36].

Domestic sheep (*Ovis aries*) are the second most abundant ruminant livestock after cattle [37] and are an important component of livestock enterprises in tick-endemic areas. Ticks transmit to sheep several pathogens, including viruses (tick-borne encephalitis virus, Thogoto virus, Louping-ill virus, Crimean-Congo haemorrhagic fever virus), bacteria (*Mycoplasma ovis*, *Anaplasma ovis*, *Borrelia burgdorferi s.l*., *Francisella tularensis*, *Dermatophilus congolensis*, *Coxiella burnetti*) and protozoa (*Babesia* spp. and *Theileria* spp.) [38–44]. Some of these pathogens cause important zoonotic diseases such as Crimean Congo Haemorrhagic fever, *Q fever* and Human granulocytic anaplasmosis [45] resulting in negative impacts on human and animal health, and significant socio-cultural and economic losses. Despite reports on individual variations in tick burdens in sheep including prevalence and infestation intensity [46–49], little is known regarding the genetic basis of this phenotype. In this study, taking advantage of observed individual natural variation in tick infestation in two indigenous sheep (Barbarine (B; fat-tailed) and Queue Fine de ľOuest (Q; thin-tailed)) from Tunisia [44], we investigated the possible genetic basis of the trait through the analysis of genome-wide 600 K SNP genotype data from 165 individuals showing high- and low-infestation (HR/LR) to ticks and piroplasm infections. Our results suggest tick resistance could be the outcome of multigene associations with *STX5* being the possible prime, and *RAB6B, TF, SLCO2A1* and *STXBP6* being the likely potential, candidate genes driving genetic variation for tick infestation in sheep.

## Results

### Population genetic structure

**Fig. 1:**
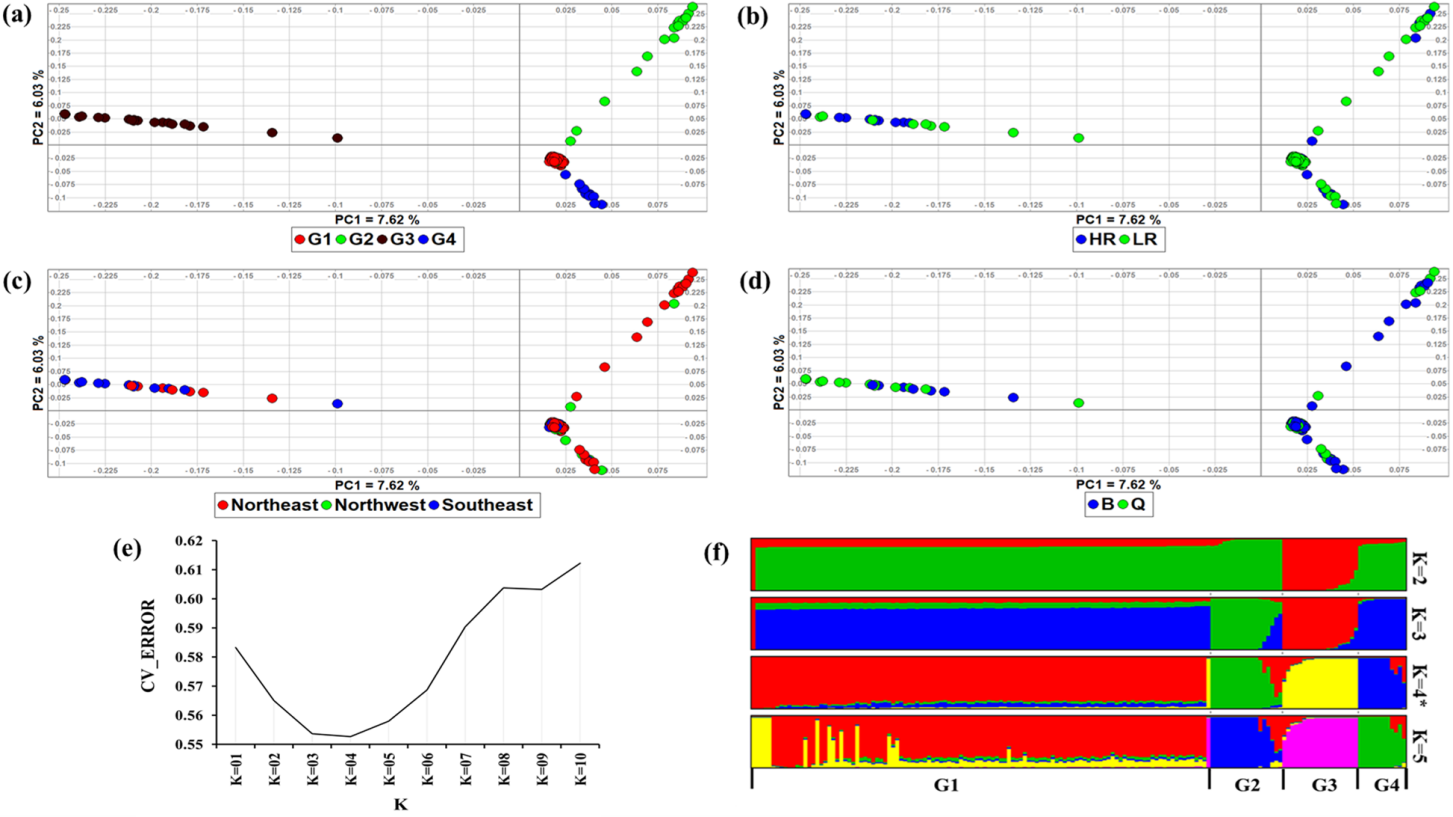
Genetic structure of the two cohorts (HR (high resistant), LR (low resistant)) of Tunisian sheep. **a**, **b**, **c** and **d** PCA cluster analysis showing PC1 and PC2; **e** Cross-validation plot for admixture analysis;**f** Admixture analysis plot showing the genetic backgrounds present in the study cohorts for 2≤*K*≤5

Genetic structure and relationship were investigated with principal component analysis (PCA) and ADMIXTURE tool. The first and second PCs of the PCA explained respectively, 7.63 and 6.04% of the total genetic variation (Fig. 1). They separated the study individuals into four genetic groups, herein named G1, G2, G3 and G4 (Fig. 1a). These four groups did not correspond to the resistance status to ticks (HR/LR) (Fig. 1b), the sampling region (northeast, northwest, southeast) (Fig. 1c) and breed (B/Q) (Fig. 1d).

Following ADMIXTURE analysis, the lowest CV error was at *K* = 4 (Fig. 1e) suggesting at least four genetic clusters in the dataset. The clusters corresponded to the four genetic groups revealed by PCA but not to the resistance status to ticks, sampling region and breed. We therefore named the four genetic clusters as G1, G2, G3 and G4 (Fig. 1f). The G1 cluster occurs in a few individuals of G2, G3 and G4, pointing to its possible introgression into the latter.

### Genome variation

Descriptive statistics, providing insights on genetic diversity were estimated for the overall dataset, the two breeds (B/Q), the two cohorts (HR/LR) and the four genetic groups (G1, G2, G3, G4) (Table 1). The overall estimates of genetic diversity represented by observed (*H*_O_) and expected (*H*_E_) heterozygosity averaged 0.3305 ± 0.0305 and 0.3470 ± 0.00006, respectively. The individual values for each statistic were in general higher than 0.3286 ± 0.0344. The mean length of ROH was 3.406 ± 0.4072 and ranged between 3.032 ± 0.5213 Mb (G1) and 3.728 ± 1.004 Mb (G4). The mean value of *F*_ROH_ was 0.0549 ± 0.0842 and ranged between 0.0239 ± 0.0527 (G1) and 0.1612 ± 0.1029 (G3). The average value of *F* was 0.0476 ± 0.0881 with a range of between -0.0866 ± 0.1339 (G4) and 0.0525 ± 0.0991 (HR). The LR cohort showed higher values of *H*_O_ and *H*_E_ compared to HR. At the opposite, the LR cohort showed lower values of *ROH* size, *F*_ROH_ and *F*. For the four genetic groups, G4 had the highest values of *H*_O_, *H*_E_ and mean length of ROH while the lowest values were observed in G1, G2 and G1, respectively. The *F*_ROH_ was highest in G3 and lowest in G1. Except G1, the other three genetic groups showed negative values of *F*.

**Table 1:** Indices of genetic diversity generated for the two breeds, two cohorts and four genetic groups of Tunisian Sheep analysed in this study

	N	H_O_ (Mean ± Sd)	H_E_ (Mean ± Sd)	ROH (Mb) (Mean ± Sd)	F_ROH_ (Mean ± Sd)	F (Mean ± Sd)
**Breed**						
Barbarine (B)	105	0.3313 ± 0.0299	0.3470 ± 0.00003	3.332 ± 0.4434	0.0515 ± 0.0826	0.0452 ± 0.0862
Queue Fine de l’Ouest (Q)	60	0.3291 ± 0.0322	0.3468 ± 0.00010	3.520 ± 0.5242	0.0625 ± 0.0881	0.0511 ± 0.0929
**Cohort**						
LR	74	0.3321 ± 0.0289	0.3477 ± 0.00007	3.377 ± 0.4750	0.0507 ± 0.0803	0.0447 ± 0.0832
HR	74	0.3286 ± 0.0344	0.3468 ± 0.00006	3.439 ± 0.4117	0.0618 ± 0.0940	0.0525 ± 0.0991
**Genetic group**						
G1	115	0.3416 ± 0.0192	0.3487 ± 0.00007	3.032 ± 0.5213	0.0239 ± 0.0527	0.0204 ± 0.0551
G2	18	0.3424 ± 0.0326	0.3331 ± 0.00003	3.667 ± 0.5966	0.1335 ± 0.0798	-0.0278 ± 0.0979
G3	20	0.3606 ± 0.0464	0.3397 ± 0.00007	3.435 ± 0.4571	0.1612 ± 0.1029	-0.0616 ± 0.1367
G4	12	0.3798 ± 0.0468	0.3495 ± 0.00002	3.728 ± 1.004	0.0850 ± 0.1105	-0.0866 ± 0.1339
**Overall**	165	0.3305 ± 0.0305	0.3470 ± 0.00006	3.406 ± 0.4072	0.0549 ± 0.0842	0.0476 ± 0.0881

**Fig. 2:**
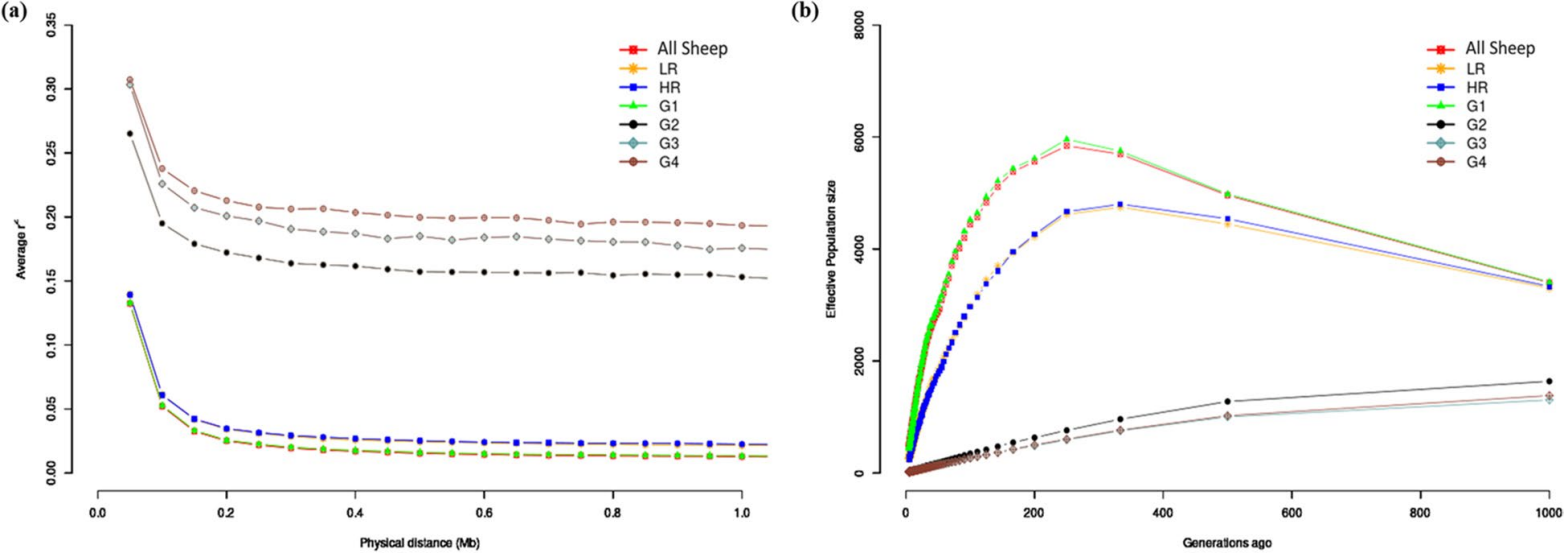
Trends in LD decay (**a**) and *N*_*e*_(**b**) across 1000 generations for the two cohorts (HR (high resistant), LR (low resistant)) and four genetic backgrounds (G1, G2, G3 and G4) of Tunisian sheep

We assessed the decay in linkage disequilibrium (LD; Fig. 2a) and the trends in effective population size (*N*_*e*_; Fig. 2b) for the overall dataset, the two cohorts (HR/LR) and the four genetic groups (G1-G4). The overall pattern of decay in LD as a function of genomic distance was the same across all the classes of datasets analysed. It generally revealed higher LD at shorter distances which decayed rapidly plateauing off around 0.2 Mb. The G2, G3 and G4 showed persistently higher *r*^2^ values (*r*^2^ > 0.15) and thus higher LD compared to the overall dataset, HR, LR and G1 (*r*^2^ < 0.15). The trends in *N*_*e*_ over generation time showed different profiles for the data classes analysed. The overall dataset and G1 group showed the highest *N*_*e*_ which increased gradually reaching maxima around 350 generations ago and then declined rapidly to the present time. The HR and LR cohorts showed the next lowest estimate of *N*_*e*_ which increased gradually up to around 350 generations ago and was followed by a rapid decline to the present. The G2, G3 and G4, had the lowest *N*_*e*_ across all generations which declined gradually to the present time.

### Genome-wide selection signature analysis

We assessed selection signatures using runs of homozygosity (ROH), Logistic regression- genome-wide association analysis (LR-GWAS), cross-population extended haplotype homozygosity (XP-EHH) and genetic differentiation (F_ST_) to gain insights on the regions of the genome correlating with resistance to ticks (HR verses LR) considering equal sample size of 74 animals in both cohorts.

**Fig. 3:**
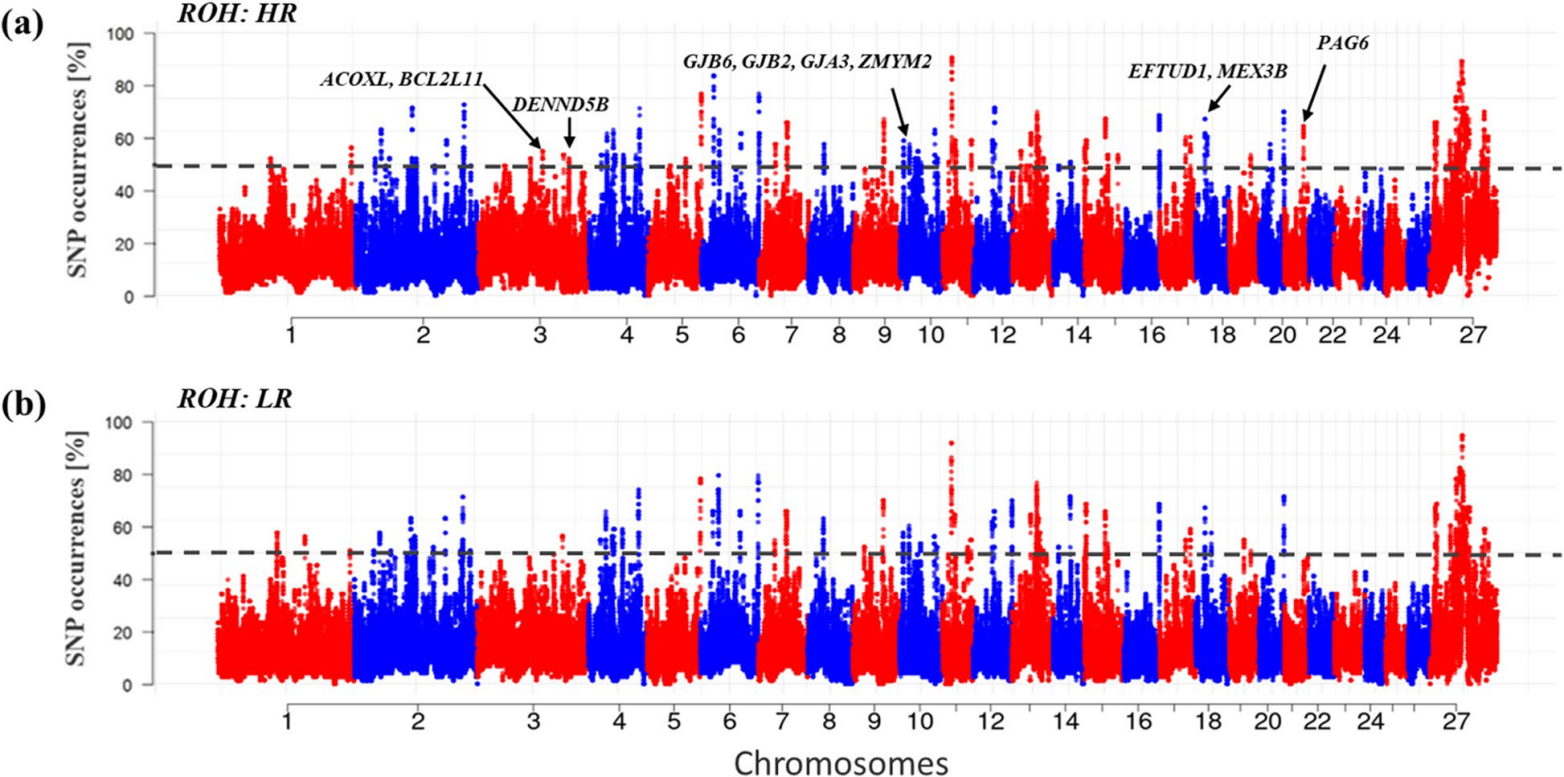
Manhattan plots showing the genome-wide distribution frequency of SNPs in stretches of ROH regions. The dashed lines indicate the 50% threshold for each cohort (HR (high resistant), LR (low resistant)) of Tunisian sheep investigated here

**Fig. 4:**
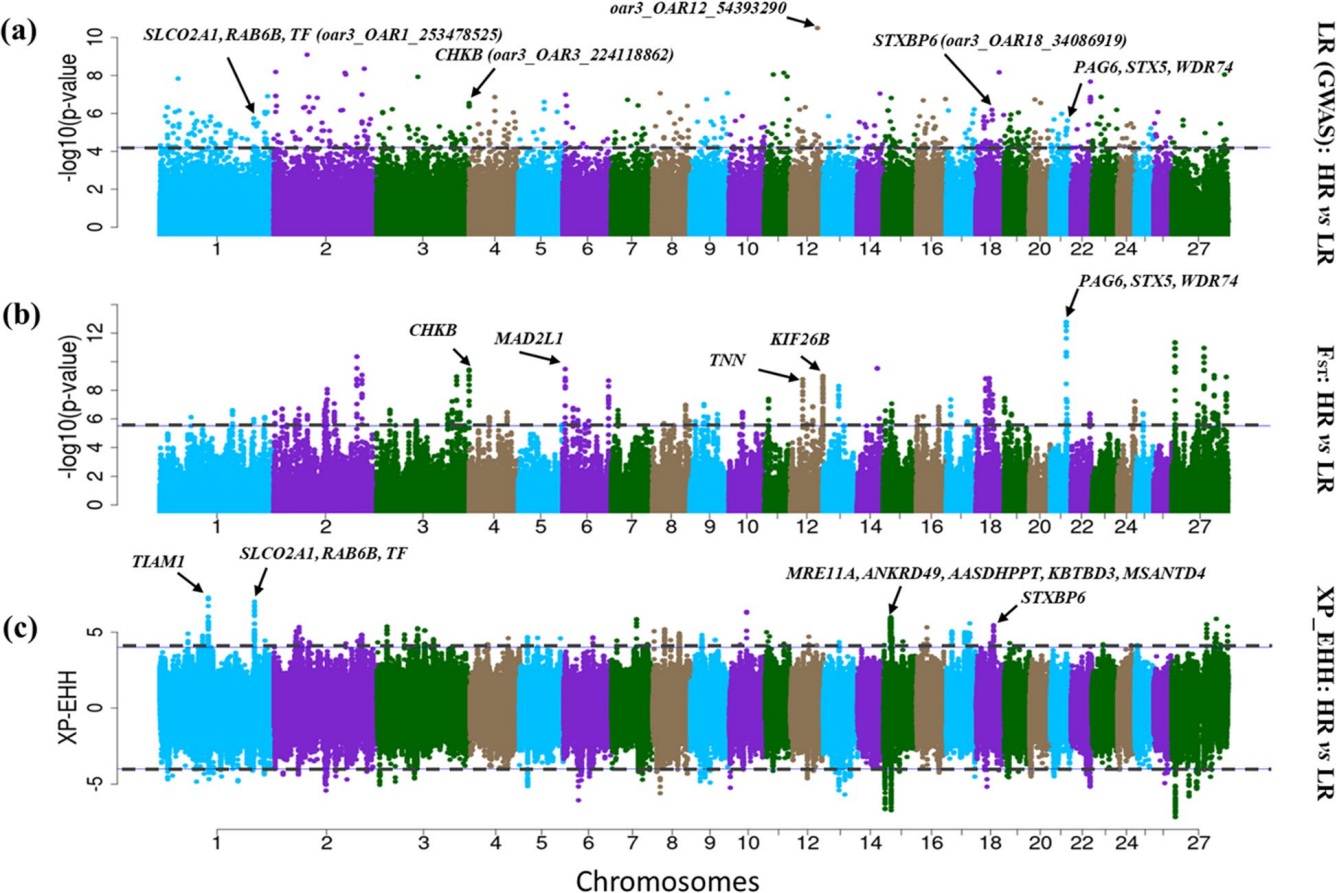
Manhattan plots showing the genome-wide distribution of SNPs following **a** LR-GWAS, **b** F_ST _and **c** XP-EHH analysis using the two cohorts (HR (high resistant), LR (low resistant)) of Tunisian sheep analysed in this study

The ROH approach identified 110 and 105 ROH regions spanning 280 and 281 annotated genes in the HR and LR cohorts (Fig. 3a and b; Supplementary Table S1, S2), respectively. The LR-GWAS identified 242 candidate regions spanning 561 genes (Fig. 4a), and F_ST_ identified 79 candidate regions, showing genetic differentiation between HR and LR (Fig. 4b), and spanning 243 genes. The XP-EHH identified 76 candidate regions spanning 187 genes (Fig. 4c). These 397 candidate regions overlapped 991 genes (Supplementary Table S3, S4, S5).

We used the ROH approach to identify cohort-specific selection signatures. We therefore compared the ROH results of HR and LR (Fig. 4a and b) and identified 30 ROH regions, spanning 57 annotated and 20 uncharacterized (prefixed “LOC”) genes that were specific to the HR cohort (Supplementary Table S6). Considering these HR-specific ROH regions and those identified by LR-GWAS, XP-EHH and F_ST_, there were 45 candidate regions that were simultaneously identified by at least two methods across 17 autosomes and spanning 160 genes of which, 56 remain uncharacterized (Table 2). Among the putative candidate genes identified in the 45 candidate regions were immune system function genes (*CDC42EP1, CD164, CD180, CDH18, MYO*), solute carriers (*SLCO2A1, SLC26A3, SLC24A3, SLC22A8*) and a chemokine receptor (*CCIN*). Of these genes, *SLCO2A1* (OAR1), *MYO* (OAR8), *SLC24A3* (OAR13), *CD180* (OAR16) and *CDH18* (OAR16) occurred closest to the most significant SNP as identified by LR-GWAS in the respective candidate regions. Four genes (*RAB6B* (OAR1), *TF* (OAR1), *SLCO2A1* (OAR1), *STXBP6* (OAR18) and *STX5* (OAR21)) found in our candidate regions are of particular interest with regard to the objectives of this study. They have been observed to be directly or indirectly associated with body weight traits in various livestock species and resistance to ticks in cattle.

**Table 2:** Candidate regions, SNPs with topmost score in LR-GWAS, and associated genes that were identified by at least two methods of selection signature analysis between the high- (HR) and low-resistant (LR) sheep cohorts

Reg	OAR	Start	Stop	Size (Mb)	Method				Top SNP (LR-GWAS)	No. of genes	Genes (Top gene^a^)	*P* value*
					ROH	LR-GWAS	F_ST_	XP-EHH				
1	1	122,490,001	122,680,000	0.190	✗	✓	✗	✓	oar3_OAR1_122581225	4	***TIAM1***, *LOC105616413, LOC105616409, LOC105616407*	3.10E-06
2	1	226,950,001	227,160,000	0.210	✗	✓	✓	✗	oar3_OAR1_227042621	5	*GFM1, LXN*, ***MLF1***, *RSRC1, LOC105603699*	2.55E-06
3	1	253,380,001	253,660,000	0.280	✗	✓	✓	✓	oar3_OAR1_253478525	4	*LOC105604371*, ***SLCO2A1***, *RAB6B, TF*	2.70E-06
4	2	51,910,001	52,140,000	0.230	✗	✓	✓	✗	oar3_OAR2_52043939	7	*RNF38*, ***GNE***, *CLTA, CCIN, GLIPR2, LOC101111253, RECK*	2.72E-06
5	2	53,110,001	53,250,000	0.140	✗	✗	✓	✓	oar3_OAR2_53171448	4	*C2H9orf131, DNAJB5*, ***KIAA1045***, *LOC101110212*	1.79E-06
6	2	62,970,001	63,160,000	0.190	✗	✓	✗	✓	oar3_OAR2_63061644	3	*ANXA1*, ***LOC105607837***, *LOC105607840*	2.68E-06
7	2	84,550,001	84,740,000	0.190	✗	✓	✗	✓	oar3_OAR2_84646297	4	***BNC2***, *LOC105608204, LOC105608202, LOC105608200*	3.47E-06
8	2	85,810,001	86,140,000	0.330	✗	✓	✗	✓	oar3_OAR2_86046574	2	***ADAMTSL1***, *TRNAC-GCA*	3.18E-06
9	2	201,150,001	201,380,000	0.230	✗	✓	✓	✓	oar3_OAR2_201281356	5	*LOC101102297, C2H2orf69, TYW5, C2H2orf47*, ***LOC105610167***	2.72E-06
10	3	183,020,001	183,260,000	0.240	✓	✓	✓	✗	oar3_OAR3_183165082	2	*TRNAC-GCA*, ***DENND5B***	2.76E-06
11	3	193,980,001	194,170,000	0.190	✗	✓	✓	✗	oar3_OAR3_194077322	1	*PDE3A*	2.65E-06
12	3	213,290,001	213,550,000	0.260	✗	✓	✓	✗	oar3_OAR3_213385724	11	*PEX26*, ***TUBA8***, *CDC42EP1, LGALS2, GGA1, SH3BP1, PDXP, LGALS1, NOL12, TRIOBP, TRNAG-CCC*	2.70E-06
13	3	224,020,001	224,277,781	0.258	✗	✓	✓	✗	oar3_OAR3_224118862	11	*NCAPH2, ODF3B, KLHDC7B, SYCE3, CPT1B*, ***CHKB***, *MAPK8IP2, ARSA, SHANK3, ACR, RABL2B*	3.35E-06
14	4	48,760,001	49,000,000	0.240	✗	✓	✓	✗	oar3_OAR4_48859309	5	*CBLL1, SLC26A3*, ***LOC105611149***, *DLD, LAMB1*	2.82E-06
15	5	44,110,001	44,300,000	0.190	✗	✓	✗	✓	oar3_OAR5_44206370	3	*LOC105607063*, ***LOC101104574***, *LOC105607061*	2.68E-06
16	5	105,810,001	106,000,000	0.190	✗	✓	✓	✗	oar3_OAR5_105908550	1	***MAN2A1***	2.56E-06
17	6	5,580,001	5,770,000	0.190	✗	✓	✓	✗	oar3_OAR6_5672674	2	*LOC101115459*, ***MAD2L1***	2.60E-06
18	8	2,520,001	2,710,000	0.190	✗	✓	✗	✓	oar3_OAR8_2618920	2	***MYO***, *IMPG1*	2.55E-06
19	8	27,780,001	27,970,000	0.190	✗	✓	✓	✓	oar3_OAR8_27875909	7	*AK9, ZBTB24, MICAL1, SMPD2*, ***PPIL6***, *POLE4, CD164*	2.72E-06
20	8	63,260,001	63,450,000	0.190	✗	✓	✗	✓	OAR8_68144751.1	1	***NHSL1***	2.59E-06
21	9	27,550,001	27,740,000	0.190	✗	✓	✗	✓	oar3_OAR9_27644689	3	***TRIB***, *LOC105611791, NSMCE2*	2.63E-06
22	9	33,820,001	34,010,000	0.190	✗	✓	✓	✗	oar3_OAR9_33912632	2	***PCMTD1***, *ST18*	2.60E-06
23	9	59,370,001	59,560,000	0.190	✗	✓	✗	✓	oar3_OAR9_59469003	1	***LOC105611701***	2.70E-06
24	11	6,640,001	6,830,000	0.190	✗	✓	✗	✓	oar3_OAR11_6733973	1	***ANKFN1***	2.82E-06
25	12	29,800,001	30,030,000	0.230	✗	✓	✓	✗	oar3_OAR12_29939034	2	***KIF26B***, *LOC105616515*	2.71E-06
26	12	54,300,001	54,490,000	0.190	✗	✓	✓	✗	oar3_OAR12_54393290	4	***TNN***, *LOC105612227, KIAA0040, LOC105612225*	2.58E-06
27	13	37,980,001	38,190,000	0.210	✗	✓	✓	✓	oar3_OAR13_38099599	2	***SLC24A3***, *LOC105616699*	2.30E-06
28	15	958,579	1,427,669	0.469	✓	✗	✗	✓	s55467.1	5	*MRE11A, ANKRD49, AASDHPPT, KBTBD3*, ***MSANTD4***	1.46E-06
29	15	5,510,001	5,720,000	0.210	✗	✓	✓	✗	oar3_OAR15_5627753	3	*MMP27*, ***MMP20***, *MMP7*	2.73E-06
30	15	17,290,001	17,690,000	0.400	✗	✓	✓	✓	oar3_OAR15_17387492	3	*NPAT*, ***ATM***, *C15H11orf65*	3.01E-06
31	15	20,560,001	20,730,000	0.170	✗	✓	✓	✓	oar3_OAR15_20686078	1	***LOC105602151***	1.71E-06
32	16	1,420,001	1,610,000	0.190	✗	✓	✓	✗	oar3_OAR16_1510505	4	***LOC105602434***, *SPDL1, LOC105602438, DOCK2*	2.68E-06
33	16	2,280,001	2,470,000	0.190	✗	✓	✓	✗	oar3_OAR16_2373107	3	*KCNIP1*, ***KCNMB1***, *LOC105602444*	2.93E-06
34	16	12,310,001	12,500,000	0.190	✗	✓	✗	✓	oar3_OAR16_12408278	4	*LOC105602490*, ***CD180***, *LOC105602489, MAST4*	2.55E-06
35	16	16,060,001	16,250,000	0.190	✗	✓	✓	✓	oar3_OAR16_16150781	1	***LOC105602500***, *LOC105602501*	3.40E-06
36	16	53,590,001	53,780,000	0.190	✗	✓	✓	✗	oar3_OAR16_53686329	2	***CDH18***, *TRNAW-CCA*	2.60E-06
37	18	23,520,001	23,880,000	0.360	✓	✓	✓	✗	oar3_OAR18_23760534	2	*EFTUD1*, ***MEX3B***	2.65E-06
38	18	33,990,001	34,180,000	0.190	✗	✓	✗	✓	oar3_OAR18_34086919	1	***STXBP6***	2.59E-06
39	18	39,710,001	40,000,000	0.290	✗	✓	✓	✗	oar3_OAR18_39823086	1	***LOC105603171***	3.14E-06
40	19	880,001	1,070,000	0.190	✗	✓	✓	✗	oar3_OAR19_977383	3	*LOC105603323, LOC105603324*, ***LOC105603325***	2.60E-06
41	19	9,910,001	10,100,000	0.190	✗	✓	✓	✗	oar3_OAR19_10008059	1	***STAC***	2.64E-06
42	21	38,480,001	38,690,000	0.210	✗	✓	✓	✗	oar3_OAR21_38598176	7	*LOC105601927, LOC101106045, LOC101104869*, ***LOC101106295***, *LOC105604093, LOC101106556, LOC101106809*	2.89E-06
43	21	38,646,061	38,906,932	0.261	✓	✓	✓	✗	oar3_OAR21_38573588	9	*LOC101106556, LOC101106809, LOC101105115, LOC101107054*, ***PAG6***, *LOC105604095, LOC443348, LOC101107317, LOC101107562*	2.83E-06
44	21	40,520,001	40,710,000	0.190	✗	✓	✓	✗	oar3_OAR21_40617710	9	*STX5, WDR74, LOC101116519, SLC3A2, CHRM1*, ***LOC101112776***, *TRNAE-UUC, LOC101117286, SLC22A8*	2.89E-06
45	25	19,550,001	19,740,000	0.190	✗	✓	✓	✗	oar3_OAR25_19649912	2	*REEP3*, ***LOC101122571***	2.60E-06
										160		

*Significant markers following LR-GWAS (Bonferroni correction *P* < 0.001); ^a^ Top genes closest to the topmost SNP as identified by LR-GWAS are in bold

Functional enrichment analysis was performed in the pool of 104 putative genes found in the 45 candidate regions that overlapped between at least one HR-specific ROH region and the ones identified by LR-GWAS, F_ST_ and XP-EHH, after excluding 56 genes that remain uncharacterized (Table 2). The analysis resulted in nine KEGG Pathways and four GO terms that were significantly enriched (Supplementary Table S7). The two top-most significant terms were Lysosome (oas04142) and microRNAs in cancer (oas05206). These terms were represented by clusters of genes (such as *GGA1*, *CD164*, and *BCL2L11*) having roles in innate immunity and disease-related inflammation [50, 51].

## Discussion

T-TBI result in negative impacts on ruminant livestock production. Therefore, the control and ultimate elimination of T-TBI should be prioritized to minimize impacts not only on animal health and production but also on human and environmental wellbeing. Large variations in susceptibility to T-TBI points to genome-wide variability that underpins inter-animal variation in tick infestation and the pathogens they transmit. Here, we generated ovine 600 K SNP genotype data, from 165 animals of two breeds of Tunisian indigenous sheep that graze natural communal pastures and with no history of anti-tick prevention intervention. The sheep comprised of individuals showing high and low resistance (HR/LR) to tick infestation and piroplasm infection [44]. We analysed the data with ROH, LR-GWAS, XP-EHH and F_ST_, to investigate signatures of selection associated with variation in tick infestation and thus possible resistance. As noted by [52], we also acknowledge that using naturally infected animals to study the genetic basis of resistance runs the risk of resistant animals being a cocktail of truly highly resistant individuals as well as those that were never exposed to infestations/infections. These factors may dilute the certainty that animals showing high or low resistance to different pathogens and parasites, could in fact be functionally dissimilar [52].

Compared to the HR, the LR cohort showed comparatively higher levels of genetic diversity but lower levels of inbreeding, though the differences were not significant (P > 0.05). The variability in the four genetic groups revealed by PCA and ADMIXTURE was of the same magnitude as that of the two cohorts. Comparable levels of genetic variation have also been reported in Egyptian [53], Algerian [54], Tunisian [55] and Russian [56] breeds of sheep.

The PCA and ADMIXTURE provided corroborating evidence suggesting the absence of genetic stratification that is consistent with the *a priori* classification of the study individuals based on their susceptibility/resistance to tick infestation/piroplasm infection, geographic sampling regions and breeds. The lack of genetic differentiation corresponding to susceptibility/resistance to tick infestation/piroplasm infection is not surprising. A similar lack of genetic stratification corresponding to *a priori* classification of sheep based on levels of prolificacy [57] or resistance to gastro-intestinal nematodes [52, 58] has been reported. [59] also observed no clear genetic differentiation between sub-populations of dual-purpose Black and White and German Holstein cattle. Our result suggests that the animals comprising the HR and LR cohorts are not highly divergent for the tick-resistance phenotype. This can be attributed to one or more of the following factors, (i) weak selection pressure driving differences in tick susceptibility/resistance and therefore any favourable alleles are likely to be rare, (ii) low level of tick burden that does not result in detectable genomic differentiation, and (iii) absence of farmer-driven preferential use of tick-resistant animals for breeding. The lack of genetic differentiation corresponding to breeds and sampling regions was not unexpected. Past and recent human-mediated translocations and dispersal of the two breeds across Tunisia has brought different sheep breeds in close geographic proximity and contact [60] resulting in cross-mating that is homogenizing their genomes. A similar finding was reported by [55]. The fact that B and Q could not be differentiated by ADMIXTURE provides evidence at the genome level that supports past and on-going mating of the fat-tailed Barbarine with thin-tailed sheep to reduce the tail-fat content in the Barbarine carcass [61].

LD was estimated for all marker pairs using the *r*^2^ metric plotted as a function of increasing genomic distance. The overall pattern of LD decay is the same for all the classes of datasets analysed. It reveals higher LD at shorter distances which declines rapidly and plateaus off at around 100 Kb. This differs from what has been observed in commercial breeds whose LD plateaus off at around 150 Kb [62] and is most likely the result of differences in breeding history. Specifically, the study populations comprised a broad genetic base while that of commercial breeds has been narrowed down by bottlenecks arising from breed formation and stringent artificial selection for economic traits. Both the HR and LR cohorts showed identical patterns of LD decay, and trends in *N*_*e*_. Although the two cohorts showed differences in tick infestation [44], this result suggests that they share aspects of their past and recent population demography and breeding history. This may be the case as their genomes showed no differentiation aligning with their classification based on tick susceptibility/resistance. We also assessed the demographic profiles of the four genetic groups underlying the genome architecture of the study individuals as revealed by PCA and ADMIXTURE. The G2, G3 and G4 genetic groups had the highest r^2^ values (r^2^ > 0.15) and thus highest LD over short genomic distances and a decay curve with persistently elevated values compared to G1. A similar pattern was observed in the primitive Soay sheep [62] and was attributed to its small effective population size due to its genetic isolation in Scotland’s St. Kilda Island. Indeed, G2, G3 and G4 show the lowest *N*_*e*_ which declines gradually over all generations. The possible reason(s) for the gradual decline in *N*_*e*_ in the three groups remains unknown and is worth investigating. In contrast, LD was lower (*r*^2^ < 0.15) in G1 across all genomic distance intervals and the group also showed the highest *N*_*e*_ over all generations. A similar pattern and magnitude of LD was reported in the Iranian Qezel sheep and was attributed to high genetic diversity in the breed [62]. This explanation may however not explain our results as there was no significant differences in the level of genetic diversity across the different classes of datasets analysed herein. Despite being unexplained, we postulate that the low LD and high *N*_*e*_ in G1, and similar magnitude of genetic diversity is unlikely to reflect any biological phenomenon.

Since millennia, ticks have parasitized animals for blood-meal to the extent of developing, through co-evolution, a sophisticated armoury that guarantees their biological success. To explore genomic signatures encoding individual variation in tick infestation and thus host resistance to ticks, we segregated Tunisian sheep into two extreme groups comprising animals showing high- and low-resistance (HR/LR) to tick infestations and piroplasm infections following the results of a previous study on the same animals [42]. The HR group comprised individuals showing neither tick infestation nor piroplasm infection, and the LR group included animals that were infested by ticks and infected by piroplasms. The differences in tick infestation/resistance phenotype between the two groups was significant [44] and their comparison was thus used to maximize the likelihood of identifying biologically meaningful and statistically significant results. We therefore performed a comparative analysis with ROH, LR-GWAS, XP-EHH and F_ST_ approaches to detect selection signatures and SNPs that are likely associated with variation and thus resistance to tick infestation.

The four approaches revealed 45 candidate regions, that overlapped between at least two approaches, and which spanned 104 characterised genes. The LR-GWAS and F_ST_ identified a selection signature on OAR21 spanning nine genes, one of which was *STX5* (Syntaxin-5). In a study of Belmont Red cattle, *STX5* was amongst 11 of 14 genes that showed a significant increase in its level of expression in the skin of animals that were highly resistant to ticks at time zero post infestation compared to animals of low resistance [63]. The expression level was more pronounced in 3-hour skin samples, suggesting a response to tick attachment which could contribute to host innate immunity and higher resistance to ticks. *STX5* regulates the endoplasmic reticulum channel-release properties of polycystin-2, a member of the transient receptor potential cation channel family that can function as an intracellular calcium (Ca^2+^) release channel [64]. Based on the increased expression levels of the 11 (most of which are Ca^2+^ dependent genes) out of 14 genes that they tested, [63] suggested that the high mRNA transcription levels of Ca^2+^ signaling genes in skin of HR animals may explain their increased resistance to ticks. Previous tick exposure may prime animals that exhibit the HR phenotype to resist further infestations via increased expression of Ca^2+^ signaling proteins and based on these results, we suggest that *STX5* could be a prime candidate gene driving tick resistance in sheep.

Environmental changes can exert positive or negative effects on mechanisms of thermoregulation that can influence tick burdens [65]. It has been observed that tick burdens in cattle might be correlated with traits that influence thermal comfort [66]. For instance, traits such as skin thickness, hair density and skin secretions can influence tick resistance and thermal comfort in domestic livestock as they affect the ability of the animal to dissipate heat [67]. Observations in Colombian cattle showed that high temperature humidity index (THI) values were associated with lower tick burdens and a higher tick infestation would be expected when animals experience higher thermal discomfort [66]. These findings are relevant to our study because one of our candidate regions that was revealed by LR-GWAS and XP-EHH on OAR18 spanned *STXBP6* (Syntaxin Binding Protein 6) and another one that was revealed by LR-GWAS, XP-EHH and F_ST_ on OAR1 spanned *SLCO2A1* (*OATP2A1*). Both genes were closest to the most significant SNP as revealed by LR-GWAS. *STXBP6* was one among a cluster of genes found to be upregulated in the testes of roosters exposed to acute heat stress [68]. Transcripts of *STXBP6* were also found to be among eight of other genes that were correlated with the modified Rodnan skin thickness score and forced vital capacity in humans suffering from scleroderma and systemic sclerosis [69], a condition that is characterized by thickening and hardening of the skin. Skin thickness is a physical barrier that can confer resistance to tick infestation(s); animals with thin skins having significantly lower tick burdens [63, 70, 71]. With the exception of birds and canids, thin skins allow animals to dissipate more heat through sweating and evaporative cooling when ambient temperatures are above thermoneutrality [65], and at the same time, it decreases tick attachment rate to the host’s skin. *SLCO2A1*, a prostaglandin transporter, maintains an increased interstitial concentration of *PGE2*, a major chemical mediator of febrile response, in the hypothalamus which plays a key role in thermoregulation [72]. The function of these genes is most likely complemented by *GNE*, the gene closest to the top-most SNP as identified by LR-GWAS on a candidate region on OAR2, that likely plays a role in adaptation to climate-mediated selective pressures in sheep [73, 74]. Taken together, this information led us to hypothesize that the *STXBP6* could have a potential pleiotropic effect on skin thickness and thermoregulation in sheep that enhances tick and heat stress tolerance. *SLCO2A1* on the other hand, can enhance tick resistance by regulating fever during infestation with T-TBI as well as thermoregulation. However, these hypotheses need to be validated with more data on appropriate phenotypes such as skin and coat characteristics.

Birth weight is the earliest available body weight trait with considerable impact on lamb survival and growth performance [75]. Our analysis revealed a candidate region on OAR1 that was identified by LR-GWAS, XP-EHH and F_ST_ that spanned among others the *RAB6B* and *TF* genes. This region was observed, following GWAS analysis, to be significantly associated with birth weight in sheep [76, 77]. Growth is essentially associated with bone development and it was found that *STXBP6* had potential pleiotropic effect on bone tissue and fecundity traits in chickens [78] and was found to be in a region under selection in broilers [79] and layers [80] suggesting that it may influence body weights. In several cases, T-TBI can destabilize host growth and development. To counter against such destabilization, we suggest that natural selection may be acting on the regions spanning *RAB6B*, *TF* and *STXBP6* to enhance growth and development stability early in life as an adaptive strategy for survival in the context of high T-TBI challenge.

## Conclusions

In this study, we investigated selection signatures in Tunisian indigenous sheep using an *a priori* defined two groups of animals presenting contrasting phenotypes of high- and low-resistance to ticks, based on tick counts and piroplasm infections. The two cohorts were characterized by similar levels of genetic variation and a fine scale genomic structure that could not be explained by tick resistance status, geographic sampling region and breeds. Four methods of detecting selection signatures identified regions of the genome that were most likely associated with differences in tick infestation and thus resistance; with our results suggesting that *STX5* could be a prime candidate driving tick resistance in sheep. We further hypothesized that *STXBP6* and *SLCO2A1* could be potential candidates for tick resistance in indigenous sheep and should be investigated further. The occurrence of *RAB6B* and *TF* in a candidate region that was significantly associated with body weight traits indicates that natural selection may be enhancing growth and development stability as an adaptive strategy to tick infestation. For these genes to qualify as candidates for enhancing genetic resistance to ticks in sheep through precision breeding (genomic selection and/or gene editing), their potential effects should be quantified through gene expression studies involving resistance and susceptible animals and identify actionable variants encoding the trait. Such quantification would benefit from the inclusion of variables, such as skin and coat characteristics, to investigate their influence on tick infestation and resistance. Overall, our findings provide a genomic reference for understanding the genetic architecture of tick resistance and cryptic divergence in indigenous sheep.

## Methods

### Study animals, sampling and genotyping

The blood samples used in this study were collected by veterinary surgeons from farmers flocks. The sampling was done during routine animal health monitoring and surveillance following standard veterinary procedures that complied with animal welfare regulations as detailed in the guidelines for the care and use of animals of the Tunisian National Council of Veterinary Surgeons (TNCVS). Prior to blood sampling, verbal permission was sought and granted by animal owners who witnessed the procedure. Therefore, permission from the Ethics Committee of the TNCVS was not required.

Two breeds, Barbarine (B) and Queue Fine de l’Ouest (Q) from Tunisia were sampled. The animals graze natural communal pastures throughout the year except during summer when they are released to forage on crop residues. Prophylactic treatment is rare, but vaccinations against Brucellosis, Bluetongue, Foot and Mouth disease and Sheep-pox are done annually by the National Veterinary Services. The animals selected for this study were sampled as part of a larger cross-sectional study on T-TBI carried out between 2018 and 2020 [44] in the northeast, northwest and southeast regions of Tunisia. The northeast and southeast regions are the homelands of the B breed although it has been dispersed throughout the country. It is the foundation of the “Tunis” and “Barbaresca” breeds found respectively, in the USA and Italy [81]. The B is also found in Libya and eastern Algeria [82]. The Q breed predominates in the northwest region although they have also been dispersed to the central, eastern and western plateaus of Tunisia. The breed is also found in mixed sheep-cropping systems in eastern Morocco and is genetically close to the Algerian Ouled Jellal [83].

For the cross-sectional study, 439 mature ewes were tagged and monitored through eight rounds of sampling as detailed by [44]. Whole blood (5 mls) was collected from each animal in EDTA coated vacutainers via jugular venipuncture and ticks were also collected from both ears, identified, counted and preserved in 70% ethanol. From the 439 ewes, 165 (B = 105; Q = 60) were purposely selected for this study based on tick scores and piroplasm infections. Genomic DNA was extracted from the blood samples with the Rapid Blood Genomic DNA Extraction Kit (Bio Basic, Markham, Canada). We used the infestation score of [84] to determine tick infestation loads as follows: 0 (no ticks), 1 (≤10), 2 (11–30), 3 (31–80), 4 (81–150), and 5 (>150), while piroplasms were detected with polymerase chain reaction [44].

From the results of population structure analysis, which showed no genetic differentiation between the two breeds, the 165 samples were assigned to two cohorts, irrespective of the breed based on extreme values of tick load and piroplasm infections viz.: (i) high tick-resistant (HR) - animals with no tick infestation(s) (score = 0) and/or no piroplasm infection(s) and (ii) low tick-resistant (LR) - animals that were highly infested with ticks (score > 81) and/or infected by piroplasms (Table 1). The DNA from the 165 samples was genotyped with the Illumina 600 K SNP BeadChip (Illumina Inc., San Diego, CA, USA) at Neogen GeneSeek, Lincoln NE, USA. The BeadChip comprises 606,006 probes targeting genome-wide SNPs, of which 577,401 are autosomal, 27,314 are on the X chromosome and 1,291 remain unassigned [85].

The raw genotypes were assessed for quality with PLINK1.9 [86] and then pruned with the following criteria: (i) one individual from a pair of highly related animals was discarded if they had an identity-by-state (IBS) score greater than 0.99, (ii) SNPs with minor allele frequencies (MAF) of no less than 0.01 were retained, (iii) individuals and SNPs with call rates lower than 90% and 95%, respectively were discarded and (iv) all unmapped SNPs were discarded. This generated a dataset of 537,214 SNPs comprising 74 HR (B = 43; Q = 31) and 96 LR (B = 65; Q = 31) individuals. This dataset was subjected to LD pruning with the parameters 50 5 0.5 representing window size, step size and *r*^2^ threshold, respectively resulting in 338,180 SNPs that were used for genetic structure analysis.

### Assessment of population genetic structure

Although the study individuals were classified *a priori* into two extreme cohorts, HR and LR, we first assessed population genetic structure and divergence to determine whether the underlying genome architecture corresponds to the two cohorts. We therefore performed principal component analysis (PCA) with PLINK v1.9 and the first two PC’s were plotted to visualize genetic relationships. We also inferred fine-scale genome structure and shared genome ancestry with the unsupervised mode of ADMIXTURE tool v1.30 [87], independent of background information on the number and frequency of alleles in the ancestral gene pool. The ADMIXTURE tool was run with a *K* range of 1-8 clusters and five replicate runs were performed for each *K* to generate cross-validation (CV) errors. The lowest CV error was used to infer the optimal number of distinct genetic clusters in the dataset.

### Assessment of genetic diversity and demographic dynamics

Expected (*H*_*E*_) and observed (*H*_*O*_) heterozygosity, patterns of LD decay across the genome and effective population size (*N*_*e*_) across generation time were investigated for each breed, the HR and LR cohorts and the genetic groups revealed by PCA and ADMIXTURE. *H*_*E*_ and *H*_*O*_ were calculated with PLINK v1.9. The extent of LD between pairwise SNPs was evaluated with the *r*^*2*^ statistic calculated with PLINK v1.9. The *N*_*e*_ over generation time was estimated with the equation *N*_*e* t_ = (1/4c) (1/*r*^*2*^ − 1) [88], where *N*_*e* t_ is the *N*_*e*_ t generations ago (t = 1/2c); *r*^2^ is the LD between pairwise SNPs; and c is the genetic distance in Morgans between pairs of SNPs.

Two coefficients of inbreeding were calculated with PLINK v1.9; the SNP based *“F*” and the runs of homozygosity (ROH) based “*F*_ROH_”. The latter was computed as the ratio of the total length of ROH to the length of autosomes (2.45 Gb) [89]. The ROHs were identified for each animal with the following parameters: (i) the minimum number of SNPs in a sliding window was set to 50; (ii) the minimum ROH length was set to 1 Mb to eliminate the impact of strong LD; (iii) each ROH spanned a minimum of 80 consecutive SNPs; (iv) one heterozygous and five missing calls per window were allowed to avoid false negative results that can arise from genotyping errors or missing genotypes; (v) the minimum SNP density was set to one SNP/100 kb, and (vi) the maximum gap between consecutive SNPs was set to 1 Mb.

### Detection of selection signatures and association analysis

To detect selection signatures spanning regions associated with genomic targets for resistance to ticks, we first investigated the distribution of ROH across the genomes of the HR and LR cohorts after standardizing the sample sizes to 74 animals per cohort. The frequency of a SNP within an ROH region was determined and a Manhattan plot visualising all the tested SNPs against their autosomal positions was generated. The most frequent SNPs occurring above the 50% cut-off threshold of the empirical distribution were taken as the most significant loci underlying an ROH under selection. To identify the ROH streatches that are associated with tick resistance, we contrasted the HR and LR ROH regions and identified the ones that were specific to HR.

We used the population differentiation statistic, F_ST_, to investigate regions of the genome showing divergence between HR and LR. The unbiased pairwise F_ST_ [90] was computed for each SNP with the R package “HIERFSTAT” [91] using a window size of 100 Kb and a window-step size of 10 Kb. Windows with less than five SNPs were excluded from the analysis. The F_ST_ values were standardized by Z transformation following [92]. To minimize the likelihood of false positive results, the windows that occurred within the top 0.001% of the normal distribution of the F_ST_ values in each chromosome and representing the most divergent regions between the two cohorts were considered putative candidates under divergent selection.

We used the software developed by [93] to estimate the unstandardized XP-EHH statistics for all SNPs, after quality control, following the comparative analysis between the HR and LR cohorts. The unstandardized XP-EHH statistics were standardized using their means and variances. We estimated the p-values of the SNPs using the standard normal distribution following findings of previous studies [94–96]. We determined the candidate regions under positive selection by clustering the significant core SNPs (*P-*value < 0.05) within a distance of less than 100 kb from the top-most SNP.

We performed the logistic regression (LR) GWAS with PLINK v1.9 to explore further, the possible genomic regions and SNPs associated with variation in tick resistance. The HR and LR cohorts were used as the test and control groups, respectively. To obtain the 99% confidence intervals for the estimated parameters, the “--ci 0.99” and “--covar” options were invoked, and Fisher’s exact test was used to generate the p-values considering age, sampling region and breed as covariates. The generated p-values were Bonferroni corrected to minimize the likelihood of false positive results. The corrected p-values were standardized and the -log10 (p-value) of 4.25 (the top 0.001) was set as the cut-off threshold to identify the candidate regions and associated SNPs. The estimations were summarized in 100 Kb window sizes and the genes and top-most SNP found within the candidate regions identified. The Manhattan plots showing the genome-wide distribution of the SNPs were generated with the R package “qqman” v3.5.1.

### Functional annotation of candidate regions

The candidate regions revealed by ROH were analysed and the ones that were specific to HR identified. These HR-specific ROH regions and the ones identified by LR-GWAS, XP-EHH and F_ST_ were analysed, and the ones that overlapped between at least two methods identified and merged using Bedtools v.2.28.0 [97]. The genes spanned by the overlapping candidate regions were retrieved using the Biomart/Ensembl (http://www.ensembl.org/biomart) tool based on the Ovine v3.1 reference genome. These set of genes were assessed for biological enrichment gene ontology (GO) and KEGG Pathway (www.kegg.jp/kegg/kegg1.html) terms compared to the full list of *O. aries* autosomal protein-coding genes with the functional annotation tool in DAVID v6.8 [98]. We further determined the functions of the putative gene from the NCBI database (http://www.ncbi.nlm.nih.gov/gene/) and review of literature.

## Supplementary Information

**Additional file 1.**

## Data Availability

The datasets generated and/or analysed during the current study are available in FIGSHARE repository, https://figshare.com/s/56deab19b63db8159c88; with the doi: 10.6084/m9.figshare.16915600.

## Abbreviations

B: Barbarine
Q: Queue Fine de l’Ouest
HR: High resistant
LR: Low resistant
PCA: Principal component analysis
T-TBI: Ticks and tick-borne infections
ROH: Runs of homozygosity
LR-GWAS: Logistic regression genome-wide association analysis
XP-EHH: cross-population extended haplotype homozygosity
F_ST_: genetic differentiation statistic
OAR: Ovine chromosome
