# Supplementary material for: Genome variation in tick infestation and cryptic divergence in Tunisian indigenous sheep
Source: BMC Genomics. 2022 Feb 28;23:167. doi: 10.1186/s12864-022-08321-1 (PMC8883713; doi:10.1186/s12864-022-08321-1)
Supplement: Supplementary file 1 — Additional file 1. [file 12864_2022_8321_MOESM1_ESM.docx]

**Supplementary Table S1.** The candidate regions spanning genes within the *HR* sheep group identified *via* ROH islands.

| **Reg.** | **Chr.** | **Start** | **Stop** | **Size (Mb)** | **No of**  **Genes** | **Genes** |
| --- | --- | --- | --- | --- | --- | --- |
| 1 | 1 | 102960696 | 102986833 | 0.026 | 1 | *NUP210L, LOC105608943* |
| 2 | 1 | 266675706 | 266761608 | 0.086 | 3 | *MORC3, CHAF1B, CLDN14* |
| 3 | 2 | 39326712 | 39364679 | 0.038 | 1 | *EBF2* |
| 4 | 2 | 51096448 | 51329779 | 0.233 | 1 | *ZCCHC7* |
| 5 | 2 | 67955833 | 68075151 | 0.119 | 1 | *LOC101108887* |
| 6 | 2 | 113842612 | 114259764 | 0.417 | 1 | *PLEKHB2* |
| 7 | 2 | 122385838 | 122512143 | 0.126 | 1 | *FSIP2* |
| 8 | 2 | 184116675 | 184224117 | 0.107 | 1 | *PTPN4* |
| 9 | 2 | 218427149 | 218677776 | 0.251 | 1 | *LOC105606769* |
| 10 | 2 | 219425968 | 219563533 | 0.138 | 4 | *USP37, VIL1, RQCD1, PLCD4* |
| 11 | 3 | 105188610 | 105221230 | 0.033 | 2 | *ACOXL, BCL2L11* |
| 12 | 3 | 129702880 | 129753390 | 0.051 | 1 | *SOCS2* |
| 13 | 3 | 172095135 | 172190994 | 0.096 | 1 | *C3H12orf42* |
| 14 | 3 | 183129985 | 183234842 | 0.105 | 1 | *DENND5B* |
| 15 | 4 | 24510293 | 24585819 | 0.076 | 1 | *LOC105612555* |
| 16 | 4 | 35347016 | 35793980 | 0.447 | 1 | *SEMA3D* |
| 17 | 4 | 48627750 | 48758013 | 0.130 | 2 | *BCAP29, SLC26A4* |
| 18 | 4 | 51613452 | 51893855 | 0.280 | 3 | *MET, CAV1, CAV2* |
| 19 | 4 | 68873688 | 68902090 | 0.028 | 5 | *HOXA3, HOXA9, HOXA7, HOXA5, HOXA6* |
| 20 | 4 | 68910285 | 68942168 | 0.032 | 2 | *HOXA2, HOXA1* |
| 21 | 4 | 68974993 | 69224698 | 0.250 | 1 | *SKAP2* |
| 22 | 4 | 70002164 | 70017866 | 0.016 | _ | *_* |
| 23 | 4 | 94343234 | 94425969 | 0.083 | 1 | *COPG2* |
| 24 | 4 | 101476571 | 101603960 | 0.127 | 2 | *CREB3L2, AKR1D1* |
| 25 | 4 | 101709270 | 101928924 | 0.220 | 1 | *TRIM24 (Forwards)* |
| 26 | 5 | 75899997 | 75941998 | 0.042 | _ | *_* |
| 27 | 5 | 107132763 | 107840616 | 0.708 | 4 | *TSLP, WDR36, CAMK4, STARD4* |
| 28 | 6 | 24689427 | 24691924 | 0.002 | 1 | *H2AFZ (Forwards)* |
| 29 | 6 | 24714766 | 24925904 | 0.211 | 4 | *DNAJB14, LAMTOR3, DAPP1, H2AFZ* |
| 30 | 6 | 36179174 | 36350660 | 0.171 | 5 | *PYURF, PIGY, LOC105615574, HERC5, HERC6* |
| 31 | 6 | 80041227 | 80256271 | 0.215 | 1 | *TECRL (Backwards)* |
| 32 | 6 | 116441509 | 116682950 | 0.241 | 7 | *FGFRL1, IDUA, SLC26A1, DGKQ, TMEM175, GAK, CPLX1* |
| 33 | 6 | 116809590 | 116996609 | 0.187 | 5 | *PCGF3, MFSD7, ATP5I, PDE6B, PIGG* |
| 34 | 7 | 32820211 | 32911371 | 0.091 | 2 | *BUB1B, PAK6* |
| 35 | 7 | 32959471 | 32960674 | 0.001 | 1 | *INAFM2* |
| 36 | 7 | 55969228 | 56175377 | 0.206 | 2 | *GLDN, CYP19* |
| 37 | 7 | 57125244 | 57310716 | 0.185 | 1 | *ATP8B4* |
| 38 | 8 | 31292323 | 31402984 | 0.111 | 1 | *PREP* |
| 39 | 8 | 31445371 | 31472292 | 0.027 | 1 | *PREP* |
| 40 | 9 | 60265603 | 60265603 | 0.000 | 1 | *RAD21* |
| 41 | 9 | 61866278 | 62074044 | 0.208 | 1 | *LOC105610478* |
| 42 | 10 | 7264485 | 7352724 | 0.088 | _ | *_* |
| 43 | 10 | 7453489 | 7510339 | 0.057 | _ | *_* |
| 44 | 10 | 18997591 | 19119971 | 0.122 | 1 | *FNDC3A* |
| 45 | 10 | 30643970 | 30721249 | 0.077 | 1 | *KATNAL1 (Forwards)* |
| 46 | 10 | 36197592 | 36262348 | 0.065 | 1 | *GJB6* |
| 47 | 10 | 36315285 | 36369348 | 0.054 | 1 | *ZMYM2* |
| 48 | 10 | 42387429 | 42387429 | 0.000 | _ | *_* |
| 49 | 10 | 70335525 | 70503476 | 0.168 | _ | *_* |
| 50 | 10 | 75261794 | 75359495 | 0.098 | 1 | *DOCK9* |
| 51 | 11 | 12276044 | 12334873 | 0.059 | 1 | *USP32* |
| 52 | 11 | 18266219 | 18586551 | 0.320 | 4 | *NF1, EVI2A, EVI2B, OMG* |
| 53 | 11 | 24602543 | 24765970 | 0.163 | 7 | *SPNS2, MYBBP1A, GGT6, TEKT1, SMTNL2, FBXO39, XAF1* |
| 54 | 11 | 26580667 | 26731105 | 0.150 | 18 | *PHF23, GABARAP, CTDNEP1, ELP5, CLDN7, SLC2A4, YBX2, EIF5A, GPS2, NEURL4, ACAP1, KCTD11, TMEM95, TNK1, PLSCR3, TMEM256, NLGN2, SPEM1* |
| 55 | 11 | 57905203 | 58032000 | 0.127 | 1 | *LOC105606322* |
| 56 | 12 | 38698936 | 38902910 | 0.204 | 1 | *TNFSF18* |
| 57 | 12 | 42767350 | 43000355 | 0.233 | 1 | *RERE* |
| 58 | 13 | 17132098 | 17218518 | 0.086 | 1 | *CCNY* |
| 59 | 13 | 37404755 | 37615042 | 0.210 | 5 | *OVOL2, PET117, CSRP2BP, ZNF133, DZANK1* |
| 60 | 13 | 48853122 | 49172111 | 0.319 | 1 | *LOC101117953* |
| 61 | 13 | 49400746 | 49990487 | 0.590 | 1 | *LOC101110438* |
| 62 | 13 | 50062652 | 50191471 | 0.129 | 1 | *HAO1* |
| 63 | 13 | 53111371 | 53156850 | 0.045 | 5 | *OPRL1, LKAAEAR1, RGS19, TCEA2, SOX18* |
| 64 | 13 | 53179250 | 53208135 | 0.029 | 3 | *PRPF6, SAMD10, ZNF512B,* |
| 65 | 13 | 53241515 | 53308183 | 0.067 | 2 | *TPD52L2, ABHD16B* |
| 66 | 13 | 53319979 | 53541061 | 0.221 | 14 | *ZBTB46, LIME1, ZGPAT, ARFRP1, TNFRSF6B, RTEL1, STMN3, GMEB2, C13H20orf195, SRMS, PTK6, PPDPF, EEF1A2, KCNQ2* |
| 67 | 13 | 62807191 | 62898310 | 0.091 | 1 | *RALY* |
| 68 | 14 | 34542168 | 34555098 | 0.013 | 5 | *RLTPR, ACD, PARD6A, ACTB, ENKD1*   \|  \| \| --- \| |
| 69 | 15 | 958579 | 1427669 | 0.469 | 5 | *MRE11A, ANKRD49, AASDHPPT, KBTBD3, MSANTD4* |
| 70 | 15 | 3615776 | 3833223 | 0.217 | 1 | *PDGFD* |
| 71 | 15 | 42202078 | 42461989 | 0.260 | 1 | *SBF2* |
| 72 | 15 | 47425386 | 47536716 | 0.111 | 1 | *LOC101104683* |
| 73 | 15 | 47781707 | 47781707 | 0.000 | 1 | *LOC101106201* |
| 74 | 15 | 67884311 | 67990507 | 0.106 | 1 | *LRRC4C* |
| 75 | 16 | 70480771 | 70966929 | 0.486 | 10 | *CCDC127, SDHA, PDCD6, AHRR, EXOC3, SLC9A3, CEP72, TPPP, BRD9, TRIP13* |
| 76 | 16 | 71407458 | 71455062 | 0.048 | 1 | *MRPL36* |
| 77 | 16 | 71525861 | 71530923 | 0.005 | 1 | *LPCAT1* |
| 78 | 17 | 52375542 | 52507494 | 0.132 | 2 | *HCAR2, KNTC1* |
| 79 | 17 | 62029896 | 62166166 | 0.136 | 2 | *RAB35, GCN1L1* |
| 80 | 18 | 19219705 | 19395763 | 0.176 | 10 | *LOC105603084, LOC101121378, LOC105603293, LOC101121632, LOC101102268, LOC101121887, LOC101122139, LOC101122387, LOC101102516, LOC101122633* |
| 81 | 18 | 23665103 | 23826989 | 0.162 | 1 | *MEX3B (Forwards)* |
| 82 | 19 | 43359084 | 43444026 | 0.085 | 1 | *SLMAP* |
| 83 | 20 | 22272855 | 22366433 | 0.094 | 2 | *PGK2, CRISP1* |
| 84 | 20 | 50038810 | 50206384 | 0.168 | 1 | *GMDS* |
| 85 | 20 | 50315944 | 50323113 | 0.007 | 1 | *GMDS* |
| 86 | 21 | 38646061 | 38906932 | 0.261 | 9 | *LOC101106556, LOC101106809, LOC101105115, LOC101107054, PAG6, LOC105604095, LOC443348, LOC101107317, LOC101107562* |
| 87 | 27 | 10429659 | 10656706 | 0.227 | 1 | *TMSB4X, LOC105605352* |
| 88 | 27 | 13253172 | 13582383 | 0.329 |  | *ZRSR2, AP1S2, GRPR, LOC105605374* |
| 89 | 27 | 41207085 | 41353781 | 0.147 | 1 | *EFHC2* |
| 90 | 27 | 41443596 | 41451579 | 0.008 | 1 | *EFHC2* |
| 91 | 27 | 47955566 | 47955566 | 0.000 | 1 | *PFKFB1* |
| 92 | 27 | 51697019 | 51748831 | 0.052 | 1 | *DGKK* |
| 93 | 27 | 51807424 | 51954092 | 0.147 | 1 | *CCNB3, DGKK* |
| 94 | 27 | 53317704 | 53624640 | 0.307 | 9 | *SLC38A5, SSX1, ZNF630, ZNF81, LOC105605481, LOC105605480, ZNF182, SPACA5, LOC105605482* |
| 95 | 27 | 53732986 | 53821358 | 0.088 | 4 | *UXT, ELK1, CFP, SYN1,* |
| 96 | 27 | 56741416 | 57545334 | 0.804 | 2 | *AR, OPHN1* |
| 97 | 27 | 57855058 | 58017052 | 0.162 | 2 | *YIPF6, OPHN1* |
| 98 | 27 | 59801858 | 60694209 | 0.892 | 18 | *KIF4A, GDPD2, LOC105605495, DLG3, TEX11, SLC7A3,SNX12, FOXO4, CXHXorf65, IL2RG, MED12, NLGN3, GJB1, LOC105605498, ZMYM3, NONO, ITGB1BP2, TAF1* |
| 99 | 27 | 62573445 | 62621379 | 0.048 | 1 | *LOC101112291* |
| 100 | 27 | 64445422 | 65059071 | 0.614 | 5 | *ATP7A, MAGT1, TRNAW-CCA, ATRX, FGF16* |
| 101 | 27 | 65775904 | 68629233 | 2.853 | 5 | *ATP7A, MAGT1, TRNAW-CCA, ATRX, FGF16* |
| 102 | 27 | 68794891 | 69211472 | 0.417 | 10 | *POU3F4, CYLC1, RPS6KA6, HDX, APOOL, ZNF711, POF1B, TRNAC-GCA, CHM, DACH2* |
| 103 | 27 | 69563113 | 74655928 | 5.093 | 1 | *PASD1* |
| 104 | 27 | 79317408 | 79459946 | 0.143 | _ | *_* |
| 105 | 27 | 105475672 | 105490793 | 0.015 | _ | *_* |
| 106 | 27 | 105556718 | 105589866 | 0.033 | _ | *_* |
| 107 | 27 | 105841683 | 105960574 | 0.119 | _ | *_* |
| 108 | 27 | 110164616 | 110554487 | 0.390 | 1 | *AGTR2* |
| 109 | 27 | 115645336 | 115660723 | 0.015 | 1 | *AMOT (Forward)* |
| 110 | 27 | 117857102 | 117991110 | 0.134 | 1 | *LOC105605621* |

**Supplementary Table S2.** The candidate regions spanning genes within the LR sheep group identified *via* ROH islands.

| **Reg.** | **Chr.** | **Start** | **Stop** | **Size (Mb)** | **No. of**  **Genes** | **Genes** |
| --- | --- | --- | --- | --- | --- | --- |
| 1 | 1 | 119257186 | 119264684 | 0.007 | 1 | *KCNE1* |
| 2 | 1 | 119352691 | 119481285 | 0.129 | 1 | *KCNE2* |
| 3 | 1 | 175397925 | 175503536 | 0.106 | 2 | *SLC9C1, CD200,* |
| 4 | 1 | 266698413 | 266716942 | 0.019 | 1 | *CHAF1B* |
| 5 | 2 | 39255231 | 39279410 | 0.024 | 1 | *LOC105607329* |
| 6 | 2 | 51113545 | 51329779 | 0.216 | 1 | *ZCCHC7* |
| 7 | 2 | 73585877 | 73596396 | 0.011 | 1 | *KIAA2026* |
| 8 | 2 | 113916007 | 114200854 | 0.285 | 1 | *LOC105608694* |
| 9 | 2 | 115133173 | 115284044 | 0.151 | _ | *_* |
| 10 | 2 | 115911162 | 115949491 | 0.038 | 1 | *HS6ST1* |
| 11 | 2 | 121868408 | 122052214 | 0.184 | _ | *_* |
| 12 | 2 | 122385838 | 122429181 | 0.043 | 1 | *FSIP2* |
| 13 | 2 | 123093673 | 123131444 | 0.038 | _ | *_* |
| 14 | 2 | 159646847 | 159706358 | 0.060 | 1 | *EPC2* |
| 15 | 2 | 184115730 | 184239544 | 0.124 | 3 | *PTPN4, EPB41L5, EPB41L5* |
| 16 | 2 | 214585680 | 214632374 | 0.047 | 1 | *SPAG16* |
| 17 | 2 | 218579911 | 218654582 | 0.075 | 1 | *TNS1* |
| 18 | 2 | 219224134 | 219335974 | 0.112 | 5 | *ARPC2, GPBAR1, AAMP, PNKD, TMBIM1* |
| 19 | 2 | 219376349 | 219590836 | 0.214 | 8 | *SLC11A1, CTDSP1, MIR26B, VIL1, USP37, RQCD1, PLCD4, ZNF142* |
| 20 | 2 | 234527737 | 234568963 | 0.041 | 1 | *KHDRBS1* |
| 21 | 3 | 172095135 | 172190994 | 0.096 | 1 | *C3H12orf42* |
| 22 | 4 | 24510293 | 24585819 | 0.076 | 1 | *LOC105612555* |
| 23 | 4 | 35194190 | 35455553 | 0.261 | 1 | *SEMA3D* |
| 24 | 4 | 48627750 | 48629109 | 0.001 | 1 | *BCAP29* |
| 25 | 4 | 48655233 | 48770377 | 0.115 | 3 | *BCAP29, SLC26A4, CBLL1* |
| 26 | 4 | 51630172 | 51906009 | 0.276 | 3 | *MET, CAV1, CAV2* |
| 27 | 4 | 68873688 | 69080875 | 0.207 | 8 | *HOXA3, HOXA9, HOXA7, HOXA5, HOXA6, HOXA2, HOXA1, HOXA4* |
| 28 | 4 | 101476571 | 101587131 | 0.111 | 2 | *CREB3L2, AKR1D1* |
| 29 | 4 | 101735579 | 102031467 | 0.296 | 1 | *TRIM24* |
| 30 | 5 | 107124030 | 107840616 | 0.717 | 4 | *TSLP, WDR36, CAMK4, STARD4* |
| 31 | 6 | 24790204 | 24925904 | 0.136 | 4 | *DNAJB14, LAMTOR3, DAPP1, H2AFZ* |
| 32 | 6 | 36050993 | 36475738 | 0.425 | 5 | *PYURF, PIGY, LOC105615574, HERC5, HERC6* |
| 33 | 6 | 80023930 | 80256271 | 0.232 | 1 | *TECRL* |
| 34 | 6 | 116440663 | 116659719 | 0.219 | 3 | *BUB1B, PAK6, PLCB2* |
| 35 | 7 | 32878388 | 32937007 | 0.059 | 7 | *FGFRL1, IDUA, SLC26A1, DGKQ, TMEM175, GAK, CPLX1* |
| 36 | 7 | 55969228 | 56221424 | 0.252 | 2 | *GLDN, CYP19* |
| 37 | 7 | 57125244 | 57324960 | 0.200 | 1 | *ATP8B4* |
| 38 | 8 | 31242591 | 31472292 | 0.230 | 1 | *PREP* |
| 39 | 9 | 23319741 | 23377366 | 0.058 | 1 | *ASAP1* |
| 40 | 9 | 60267184 | 60284353 | 0.017 | 1 | *RAD21* |
| 41 | 9 | 61904288 | 61997929 | 0.094 | 1 | *LOC105610478* |
| 42 | 10 | 7264485 | 7352349 | 0.088 | _ | *_* |
| 43 | 10 | 7453489 | 7530364 | 0.077 | _ | *_* |
| 44 | 10 | 18996973 | 19157373 | 0.160 | 1 | *FNDC3A* |
| 45 | 10 | 19174737 | 19180373 | 0.006 | 1 | *MLNR* |
| 46 | 10 | 42314372 | 42527182 | 0.213 | _ | *_* |
| 47 | 10 | 70379733 | 70503476 | 0.124 | 1 | *LOC101106781* |
| 48 | 10 | 75261794 | 75357987 | 0.096 | 1 | *DOCK9* |
| 49 | 11 | 18279677 | 18586551 | 0.307 | 4 | *NF1, EVI2A, EVI2B, OMG* |
| 50 | 11 | 24642765 | 24765970 | 0.123 | 7 | *SPNS2, MYBBP1A, GGT6, TEKT1, SMTNL2, FBXO39, XAF1* |
| 51 | 11 | 51340332 | 51359666 | 0.019 | 1 | *RNF213* |
| 52 | 11 | 57913799 | 58024052 | 0.110 | 1 | *LOC105606322* |
| 53 | 12 | 38708880 | 38902910 | 0.194 | 1 | *TNFSF18* |
| 54 | 12 | 42794226 | 43000355 | 0.206 | 1 | *RERE* |
| 55 | 12 | 78555727 | 78994587 | 0.439 | 7 | *TNNI1, LAD1, TNNT2, IPO9, SHISA4, LMOD1, TIMM17A* |
| 56 | 13 | 37404755 | 37615042 | 0.210 | 5 | *OVOL2, PET117, CSRP2BP, ZNF133, DZANK1* |
| 57 | 13 | 48853122 | 49172111 | 0.319 | 1 | *LOC101117953* |
| 58 | 13 | 49290888 | 50252882 | 0.962 | 2 | *HAO1, ADRA1D* |
| 59 | 13 | 53035139 | 53208135 | 0.173 | 9 | *NPBWR2, OPRL1, LKAAEAR1, RGS19, TCEA2, SOX18, PRPF6, SAMD10, ZNF512B* |
| 60 | 13 | 53324888 | 53493610 | 0.169 | 14 | *ZBTB46, LIME1, ZGPAT, ARFRP1, TNFRSF6B, RTEL1, STMN3, GMEB2, C13H20orf195, SRMS, PTK6, PPDPF, EEF1A2, KCNQ2* |
| 61 | 13 | 56316210 | 56515889 | 0.200 | 2 | *EDN3, ZNF831* |
| 62 | 13 | 62819952 | 62898310 | 0.078 | 1 | *RALY* |
| 63 | 14 | 10697127 | 10777298 | 0.080 | 1 | *LOC105616836* |
| 64 | 14 | 34416650 | 34566935 | 0.150 | 11 | *ATP6V0D1, AGRP, FAM65A, CTCF, RLTPR, ACD, PARD6A, ACTB, ENKD1, C14H16orf86, GFOD2* |
| 65 | 15 | 2839465 | 2901026 | 0.062 | 1 | *LOC101117272* |
| 66 | 15 | 3579025 | 3887998 | 0.309 | 1 | *PDGFD* |
| 67 | 15 | 42079219 | 42461989 | 0.383 | 1 | *SBF2* |
| 68 | 15 | 47765025 | 47781707 | 0.017 | 1 | *LOC101105946* |
| 69 | 16 | 70482123 | 70966929 | 0.485 | 10 | *CCDC127, SDHA, PDCD6, AHRR, EXOC3, SLC9A3, CEP72, TPPP, BRD9, TRIP13* |
| 70 | 16 | 71525861 | 71530923 | 0.005 | 1 | *LPCAT1* |
| 71 | 17 | 52404371 | 52498766 | 0.094 | 2 | *HCAR2, KNTC1* |
| 72 | 17 | 62031439 | 62166166 | 0.135 | 2 | *RAB35, GCN1L1* |
| 73 | 18 | 19219705 | 19395896 | 0.176 | 10 | *LOC105603084, LOC101121378, LOC105603293, LOC101121632, LOC101102268, LOC101121887, LOC101122139, LOC101122387, LOC101102516, LOC101122633* |
| 74 | 18 | 32287698 | 32331219 | 0.044 | 1 | *SIN3A* |
| 75 | 18 | 32352543 | 32451505 | 0.099 | 3 | *NEIL1, COMMD4, MAN2C1* |
| 76 | 19 | 29667019 | 29769466 | 0.102 | 1 | *LOC105603442* |
| 77 | 19 | 43431855 | 43431855 | 0.000 | 1 | *SLMAP* |
| 78 | 20 | 50021678 | 50323113 | 0.301 | 1 | *GMDS* |
| 79 | 27 | 10429659 | 10656083 | 0.226 | 1 | *TMSB4X, LOC105605352* |
| 80 | 27 | 13273025 | 13582383 | 0.309 | 4 | *ZRSR2, AP1S2, GRPR, LOC105605374* |
| 81 | 27 | 41215320 | 41353781 | 0.138 | 1 | *EFHC2* |
| 82 | 27 | 51705470 | 51954092 | 0.249 | 1 | *CCNB3, DGKK* |
| 83 | 27 | 53341428 | 53619641 | 0.278 | 9 | *SLC38A5, SSX1, ZNF630, ZNF81, LOC105605481, LOC105605480, ZNF182, SPACA5, LOC105605482* |
| 84 | 27 | 53732986 | 53751604 | 0.019 | 1 | *LOC101116635* |
| 85 | 27 | 53756683 | 53809441 | 0.053 | 4 | *UXT, ELK1, CFP, SYN1,* |
| 86 | 27 | 56765431 | 57184172 | 0.419 | 2 | *AR, OPHN1* |
| 87 | 27 | 57291292 | 57291292 | 0.000 | 2 | *YIPF6, OPHN1* |
| 88 | 27 | 57808129 | 57882770 | 0.075 | 1 | *OPHN1* |
| 89 | 27 | 59656932 | 59690768 | 0.034 | 2 | *PDZD11, DGAT2L6* |
| 90 | 27 | 59695972 | 60245496 | 0.550 | 11 | *PDZD11, AWAT1, P2RY4, ARR3, KIF4A, LOC105605496, GDPD2, LOC105605495, DLG3, TEX11, SLC7A3* |
| 91 | 27 | 60305507 | 60682186 | 0.377 | 18 | *KIF4A, GDPD2, LOC105605495, DLG3, TEX11, SLC7A3,SNX12, FOXO4, CXHXorf65, IL2RG, MED12, NLGN3, GJB1, LOC105605498, ZMYM3, NONO, ITGB1BP2, TAF1* |
| 92 | 27 | 62493897 | 62639839 | 0.146 | 1 | *LOC101112291* |
| 93 | 27 | 62863334 | 62863334 | 0.000 | 1 | *LOC101112542* |
| 94 | 27 | 63002879 | 63139945 | 0.137 | 1 | *SLC16A2* |
| 95 | 27 | 64445422 | 65107031 | 0.662 | 5 | *ATP7A, MAGT1, TRNAW-CCA, ATRX, FGF16* |
| 96 | 27 | 65775904 | 68589071 | 2.813 | 12 | *ZCCHC5, LPAR4, P2RY10, GPR174, TRNAW-CCA, ITM2A, LOC101119617, TBX22, FAM46D, BRWD3, HMGN5, SH3BGRL* |
| 97 | 27 | 70614867 | 71450851 | 0.836 | 2 | *CYLC1, RPS6KA6* |
| 98 | 27 | 72002213 | 73167971 | 1.166 | 3 | *APOOL, POF1B, ZNF711* |
| 99 | 27 | 73420647 | 73731000 | 0.310 | 1 | *CHM* |
| 100 | 27 | 74181490 | 74655928 | 0.474 | 1 | *DACH2* |
| 101 | 27 | 79239980 | 79239980 | 0.000 | 1 | *CNGA2 (Forwards)* |
| 102 | 27 | 79287073 | 79447883 | 0.161 | 3 | *PRRG3, PASD1, FATE1* |
| 103 | 27 | 105556718 | 105589866 | 0.033 | _ | *_* |
| 104 | 27 | 110202695 | 110450412 | 0.248 | 1 | *AGTR2* |
| 105 | 27 | 117899094 | 117950800 | 0.052 | 1 | *CHRDL1* |

**Supplementary Table S3**. The candidate regions spanning genes within the HR (HR vs LR) sheep group identified via LR-GWAS.

| **Reg.** | **Chr.** | **Start** | **Stop** | **Size (Mb)** | **No of**  **Genes** | **Genes** |
| --- | --- | --- | --- | --- | --- | --- |
| 1 | 1 | 620001 | 810000 | 0.190 | 1 | *C1H2orf54* |
| 2 | 1 | 17790001 | 17990000 | 0.200 | 6 | *MPL, CDC20, ELOVL1, MED8, LOC101107405, HYI* |
| 3 | 1 | 18490001 | 18680000 | 0.190 | 2 | *KLF17, DMAP1* |
| 4 | 1 | 19320001 | 19510000 | 0.190 | 4 | *EIF2B3, HECTD3, UROD, ZSWIM5* |
| 5 | 1 | 31110001 | 31380000 | 0.270 | 3 | *C8A, C8B, DAB1* |
| 6 | 1 | 36490001 | 36680000 | 0.190 | 2 | *TM2D1, PATJ* |
| 7 | 1 | 43440001 | 43640000 | 0.200 | 1 | *WLS* |
| 8 | 1 | 45600001 | 45940000 | 0.340 | 4 | *LRRC40, SRSF11, ANKRD13C, CTH* |
| 9 | 1 | 65260001 | 65460000 | 0.200 | 2 | *PKN2, LOC101119517* |
| 10 | 1 | 65580001 | 65770000 | 0.190 | 1 | *LOC101119773* |
| 11 | 1 | 69330001 | 69600000 | 0.270 | 3 | *MTF2, TMED5, CCDC18* |
| 12 | 1 | 75180001 | 75370000 | 0.190 | 1 | *SNX7* |
| 13 | 1 | 77050001 | 77250000 | 0.200 | 2 | *LOC101113636, LOC101114226* |
| 14 | 1 | 96640001 | 96830000 | 0.190 | 1 | *SEC22B* |
| 15 | 1 | 105090001 | 105290000 | 0.200 | 12 | *IQGAP3, TTC24, APOA1BP, GPATCH4, HAPLN2, BCAN, NES, CRABP2, ISG20L2, RRNAD1, MRPL24, HDGF* |
| 16 | 1 | 111860001 | 112050000 | 0.190 | 2 | *C1H1orf111, SH2D1B* |
| 17 | 1 | 116880001 | 117070000 | 0.190 | 3 | *POGK, TADA1, ILDR2* |
| 18 | 1 | 122490001 | 122680000 | 0.190 | 1 | *TIAM1* |
| 19 | 1 | 123560001 | 123750000 | 0.190 | 3 | *LOC101106046, LOC101115597, LOC101116626* |
| 20 | 1 | 123850001 | 124040000 | 0.190 | 2 | *CLDN8, cldn17* |
| 21 | 1 | 168760001 | 168950000 | 0.190 | 1 | *CBLB* |
| 22 | 1 | 170070001 | 170380000 | 0.310 | 1 | *LOC101116441* |
| 23 | 1 | 171980001 | 172170000 | 0.190 | 7 | *ARHGAP31, TMEM39A, POGLUT1, TIMMDC1, CD80, ADPRH, PLA1A* |
| 24 | 1 | 182430001 | 182670000 | 0.240 | 7 | *ARHGAP31, TMEM39A, POGLUT1, TIMMDC1, CD80, ADPRH, PLA1A* |
| 25 | 1 | 197820001 | 198010000 | 0.190 | 3 | *BCL6, RTP2, SST* |
| 26 | 1 | 226950001 | 227160000 | 0.210 | 3 | *GFM1, LXN, MLF1* |
| 27 | 1 | 233430001 | 233620000 | 0.190 | 3 | *MBNL1, WWTR1, TM4SF4* |
| 28 | 1 | 253380001 | 253660000 | 0.280 | 4 | *SLCO2A1, TF, RAB6B NPHP3* |
| 29 | 1 | 254810001 | 255000000 | 0.190 | 2 | *UBA5, NPHP3* |
| 30 | 1 | 257360001 | 257710000 | 0.350 | 7 | *PSMG1, BRWD1, LOC105604538, HMGN1, WRB, LCA5L, SH3BGR* |
| 31 | 1 | 259380001 | 259570000 | 0.190 | 1 | *BACE2* |
| 32 | 1 | 260260001 | 260450000 | 0.190 | 2 | *C2CD2, UMODL1* |
| 33 | 2 | 6530001 | 6720000 | 0.190 | 1 | *ASTN2* |
| 34 | 2 | 17320001 | 17510000 | 0.190 | 2 | *TAL2, FKTN, FSD1L* |
| 35 | 2 | 51910001 | 52140000 | 0.230 | 5 | *RNF38, GNE, CCIN, GLIPR2, RECK* |
| 36 | 2 | 62970001 | 63160000 | 0.190 | 1 | *ANXA1* |
| 37 | 2 | 71890001 | 72080000 | 0.190 | 1 | *GLIS3* |
| 38 | 2 | 76220001 | 76410000 | 0.190 | 1 | *PTPRD* |
| 39 | 2 | 80470001 | 80660000 | 0.190 | 1 | *TYRP1* |
| 40 | 2 | 84550001 | 84740000 | 0.190 | 1 | *BNC2* |
| 41 | 2 | 88460001 | 88650000 | 0.190 | 1 | *FOCAD* |
| 42 | 2 | 116650001 | 116880000 | 0.230 | 4 | *WDR33, LIMS2, MYO7B, IWS1* |
| 43 | 2 | 120770001 | 120960000 | 0.190 | 2 | *TFPI, CALCRL* |
| 44 | 2 | 134720001 | 134940000 | 0.220 | 1 | *OLA1* |
| 45 | 2 | 134950001 | 135140000 | 0.190 | 1 | *SP3* |
| 46 | 2 | 149580001 | 149770000 | 0.190 | 1 | *DAPL1* |
| 47 | 2 | 172410001 | 172880000 | 0.470 | 1 | *THSD7B* |
| 48 | 2 | 174090001 | 174280000 | 0.190 | 1 | *ZRANB3* |
| 49 | 2 | 177690001 | 177880000 | 0.190 | 1 | *SLC35F5* |
| 50 | 2 | 201160001 | 201380000 | 0.220 | 3 | *C2H2orf69, TYW5, MAIP1* |
| 51 | 2 | 218990001 | 219180000 | 0.190 | 1 | *RUFY4* |
| 52 | 2 | 219820001 | 220030000 | 0.210 | 4 | *WNT10A, FEV, CFAP65, IHH* |
| 53 | 2 | 226890001 | 227140000 | 0.250 | 1 | *NYAP2* |
| 54 | 2 | 234060001 | 234250000 | 0.190 | 4 | *RBBP4, ZBTB8OS, ZBTB8A, ZBTB8B* |
| 55 | 3 | 6120001 | 6310000 | 0.190 | 1 | *HMCN2* |
| 56 | 3 | 19620001 | 19810000 | 0.190 | 2 | *NOL10, PDIA6* |
| 57 | 3 | 31580001 | 31770000 | 0.190 | 7 | *WDCP, SF3B6, TP53I3, FAM228B, PFN4, FAM228A, ITSN2* |
| 58 | 3 | 37900001 | 38090000 | 0.190 | 3 | *EHD3, SNRPG, PCYOX1, TIA1* |
| 59 | 3 | 78090001 | 78280000 | 0.190 | 1 | *PRKCE* |
| 60 | 3 | 80530001 | 80720000 | 0.190 | 1 | *THADA* |
| 61 | 3 | 94970001 | 95160000 | 0.190 | 1 | *SFXN5* |
| 62 | 3 | 99920001 | 100180000 | 0.260 | 1 | *RFX8* |
| 63 | 3 | 100730001 | 100920000 | 0.190 | 3 | *PDCL3, NMS, CHST10* |
| 64 | 3 | 134810001 | 135000000 | 0.190 | 6 | *DAZAP2, POU6F1, TFCP2, CSRNP2, LETMD1, DYRK2* |
| 65 | 3 | 151900001 | 152090000 | 0.190 | 1 | *DYRK2* |
| 66 | 3 | 154030001 | 154220000 | 0.190 | 1 | *MSRB3* |
| 67 | 3 | 166060001 | 166250000 | 0.190 | 1 | *NEDD1* |
| 68 | 3 | 179100001 | 179300000 | 0.200 | 1 | *RBFOX2* |
| 69 | 3 | 183020001 | 183260000 | 0.240 | 1 | *DENND5B* |
| 70 | 3 | 193000001 | 193190000 | 0.190 | 1 | *ABCC9* |
| 71 | 3 | 193980001 | 194170000 | 0.190 | 1 | *PDE3A* |
| 72 | 3 | 213290001 | 213550000 | 0.260 | 8 | *TUBA8, CDC42EP1, LGALS2, GGA1, SH3BP1, PDXP, NOL12, LOC101119130* |
| 73 | 3 | 214580001 | 214770000 | 0.190 | 2 | *PDGFB, RPL3* |
| 74 | 3 | 215070001 | 215260000 | 0.190 | 2 | *ENTHD1, GRAP2* |
| 75 | 3 | 224020001 | 224277781 | 0.258 | 10 | *NCAPH2, LOC101121590, KLHDC7B, SYCE3, CPT1B, CHKB, MAPK8IP2, ARSA, ACR, RABL2B* |
| 76 | 4 | 13330001 | 13520000 | 0.190 | 1 | *SLC25A13* |
| 77 | 4 | 15060001 | 15250000 | 0.190 | 1 | *C1GALT1* |
| 78 | 4 | 22340001 | 22530000 | 0.190 | 1 | *DGKB* |
| 79 | 4 | 29280001 | 29470000 | 0.190 | 1 | *ABCB5* |
| 80 | 4 | 47310001 | 47500000 | 0.190 | 1 | *NAMPT* |
| 81 | 4 | 48760001 | 49000000 | 0.240 | 1 | *CBLL1, SLC26A3, DLD, LAMB1* |
| 82 | 4 | 50050001 | 50240000 | 0.190 | 1 | *STARD3NL* |
| 83 | 4 | 85690001 | 85880000 | 0.190 | 2 | *WNT16* |
| 84 | 4 | 93820001 | 94060000 | 0.240 | 4 | *ZC3HC1, KLHDC10, TMEM209, SSMEM1* |
| 85 | 4 | 107180001 | 107370000 | 0.190 | 1 | *TPK1* |
| 86 | 4 | 111220001 | 111420000 | 0.200 | 1 | *CUL1* |
| 87 | 5 | 3650001 | 3840000 | 0.190 | 7 | *SUGP1, TM6SF2, HAPLN4, NCAN, NR2C2AP, RFXANK, BORCS8* |
| 88 | 5 | 57850001 | 58060000 | 0.210 | 2 | *SH3TC2, ABLIM3* |
| 89 | 5 | 62500001 | 62750000 | 0.250 | 1 | *GRIA1* |
| 90 | 5 | 105810001 | 106000000 | 0.190 | 1 | *MAN2A1* |
| 91 | 6 | 5580001 | 5770000 | 0.190 | 1 | *MAD2L1* |
| 92 | 6 | 6310001 | 6500000 | 0.190 | 1 | *SYNPO2* |
| 93 | 6 | 6600001 | 6790000 | 0.190 | 1 | *SEC24D* |
| 94 | 6 | 16660001 | 16850000 | 0.190 | 3 | *ETNPPL, OSTC, RPL34* |
| 95 | 6 | 23790001 | 23980000 | 0.190 | 1 | *PPP3CA* |
| 96 | 6 | 107980001 | 108170000 | 0.190 | 1 | *RAB28* |
| 97 | 7 | 9870001 | 10310000 | 0.440 | 1 | *LOC101106776* |
| 98 | 7 | 10120001 | 10310000 | 0.190 | 4 | *AGGF1, BHMT, JMY, HOMER1* |
| 99 | 7 | 39560001 | 39750000 | 0.190 | 2 | *LRR1, RPL36AL* |
| 100 | 7 | 47580001 | 47770000 | 0.190 | 2 | *GTF2A2, GCNT3* |
| 101 | 7 | 57810001 | 58000000 | 0.190 | 2 | *FGF7, GALK2* |
| 102 | 7 | 62230001 | 62420000 | 0.190 | 3 | *SHF, DUOXA1, DUOX2, SORD* |
| 103 | 7 | 64980001 | 65170000 | 0.190 | 1 | *KTN1* |
| 104 | 7 | 81270001 | 81460000 | 0.190 | 2 | *DCAF4, ZFYVEL* |
| 105 | 8 | 2520001 | 2710000 | 0.190 | 9 | *MYO6, IMPG1, FIG4, AK9, ZBTB24, MICAL1, SMPD2, PPIL6, CD164* |
| 106 | 8 | 53270001 | 53460000 | 0.190 | 1 | *PTPRK* |
| 107 | 8 | 63260001 | 63450000 | 0.190 | 1 | *NHSL1* |
| 108 | 8 | 66650001 | 66840000 | 0.190 | 1 | *ADGRG6* |
| 109 | 8 | 67970001 | 68160000 | 0.190 | 1 | *PHACTR2* |
| 110 | 9 | 27550001 | 27740000 | 0.190 | 1 | *NSMCE2* |
| 111 | 9 | 33820001 | 34010000 | 0.190 | 2 | *PCMTD1, ST18* |
| 112 | 9 | 51050001 | 51240000 | 0.190 | 1 | *CRISPLD1* |
| 113 | 9 | 57200001 | 57420000 | 0.220 | 1 | *FABP5* |
| 114 | 9 | 59370001 | 59560000 | 0.190 | 1 | *EXT1* |
| 115 | 9 | 61580001 | 61770000 | 0.190 | 1 | *TRPS1* |
| 116 | 9 | 75410001 | 75600000 | 0.190 | 1 | *GRHL2* |
| 117 | 9 | 79270001 | 79470000 | 0.200 | 1 | *TSPYL5* |
| 118 | 9 | 82640001 | 82830000 | 0.190 | 1 | *CDH17* |
| 119 | 9 | 89190001 | 89380000 | 0.190 | 1 | *ATP6V0D2* |
| 120 | 10 | 32230001 | 32420000 | 0.190 | 1 | *FLT3* |
| 121 | 10 | 53400001 | 53590000 | 0.190 | 1 | *EDNRB* |
| 122 | 10 | 85170001 | 85580000 | 0.410 | 1 | *TUBGCP3* |
| 123 | 11 | 6640001 | 6830000 | 0.190 | 1 | *ANKFN1* |
| 124 | 11 | 11370001 | 11560000 | 0.190 | 1 | *BCAS3* |
| 125 | 11 | 16890001 | 17080000 | 0.190 | 2 | *TMEM98, MYO1D* |
| 126 | 11 | 19290001 | 19480000 | 0.190 | 7 | *LK, TMEM97, IFT20, TNFAIP1, POLDIP2,TMEM199, VTN* |
| 127 | 11 | 20730001 | 20920000 | 0.190 | 3 | *NSRP1, EFCAB5, SLC6A4* |
| 128 | 11 | 27320001 | 27510000 | 0.190 | 9 | *ALOXE3, PER1, TMEM107, AURKB, CTC1, PFAS, RANGRF, SLC25A35, ARHGEF15* |
| 129 | 11 | 36930001 | 37120000 | 0.190 | 2 | *B4GALNT2, GPM6A* |
| 130 | 11 | 46290001 | 46480000 | 0.190 | 2 | *METTL2A, TLK2* |
| 131 | 11 | 54330001 | 54520000 | 0.190 | 2 | *UBE2O, SPHK1* |
| 132 | 11 | 55480001 | 55670000 | 0.190 | 6 | *RNF157, OTOP3, FDXR, GRIN2C, NAT9, SLC9A3R1* |
| 133 | 12 | 17940001 | 18130000 | 0.190 | 1 | *ESRRG* |
| 134 | 12 | 21640001 | 21830000 | 0.190 | 5 | *ESRRG, EPRS, BPNT1, IARS2, RAB3GAP2* |
| 135 | 12 | 22270001 | 22520000 | 0.250 | 3 | *MARK1, C12H1orf115, LOC101123395* |
| 136 | 12 | 34440001 | 34630000 | 0.190 | 1 | *DPT* |
| 137 | 12 | 52010001 | 52200000 | 0.190 | 1 | *PDPN* |
| 138 | 12 | 54300001 | 54490000 | 0.190 | 1 | *TNN* |
| 139 | 12 | 58510001 | 58700000 | 0.190 | 2 | *TOR3A, ABL2* |
| 140 | 12 | 65440001 | 65630000 | 0.190 | 4 | *PRG4, TPR, , C12H1orf27, PDC* |
| 141 | 13 | 14800001 | 14990000 | 0.190 | 1 | *CELF2* |
| 142 | 13 | 57280001 | 57470000 | 0.190 | 2 | *RAB22A, ANKRD60* |
| 143 | 13 | 64470001 | 64660000 | 0.190 | 2 | *RBM39, PHF20* |
| 144 | 14 | 1550001 | 1740000 | 0.190 | 2 | *FA2H, WDR59* |
| 145 | 14 | 17910001 | 18100000 | 0.190 | 3 | *CNEP1R1, HEATR3, PAPD5* |
| 146 | 14 | 21280001 | 21470000 | 0.190 | 3 | *RBL2, AKTIP, RPGRIP1L* |
| 147 | 14 | 38240001 | 38430000 | 0.190 | 4 | *DHX38, TXNL4B, LOC101102413, PKD1L3* |
| 148 | 14 | 54860001 | 55050000 | 0.190 | 12 | *CD37, TEAD2, DKKL1, CCDC155, SLC17A7, PIH1D1, ALDH16A1, RPS11, FCGRT, RCN3, NOSIP, PRRG2* |
| 149 | 14 | 58660001 | 58850000 | 0.190 | 3 | *LOC101115648, MYADM, PRKCG* |
| 150 | 15 | 5510001 | 5720000 | 0.210 | 3 | *MMP27, MMP20, MMP7* |
| 151 | 15 | 15240001 | 15430000 | 0.190 | 1 | *PIWIL4* |
| 152 | 15 | 17290001 | 17600000 | 0.310 | 3 | *NPAT, ATM, C15H11orf65* |
| 153 | 15 | 21560001 | 21750000 | 0.190 | 6 | *ALG9, FDXACB1, C15H11orf1, CRYAB, C15H11orf52, DIXDC1* |
| 154 | 15 | 23160001 | 23350000 | 0.190 | 3 | *NCAM1, TTC12, DRD2* |
| 155 | 15 | 26780001 | 26970000 | 0.190 | 2 | *BUD13, ZPR1* |
| 156 | 15 | 28060001 | 28250000 | 0.190 | 2 | *TMPRSS13, IL10RA* |
| 157 | 15 | 32870001 | 33060000 | 0.190 | 2 | *UBASH3B, CRTAM* |
| 158 | 15 | 40410001 | 40600000 | 0.190 | 1 | *USP47* |
| 159 | 15 | 74740001 | 74930000 | 0.190 | 4 | *AMBRA1, HARBI1, ATG13, F2, CKAP5* |
| 160 | 15 | 75980001 | 76170000 | 0.190 | 2 | *CKAP5, PTPRJ* |
| 161 | 16 | 1420001 | 1610000 | 0.190 | 1 | *SPDL1* |
| 162 | 16 | 2280001 | 2470000 | 0.190 | 1 | *KCNMB1* |
| 163 | 16 | 12310001 | 12500000 | 0.190 | 2 | *CD180, MAST4* |
| 164 | 16 | 20480001 | 20680000 | 0.200 | 1 | *RAB3C* |
| 165 | 16 | 21890001 | 22080000 | 0.190 | 1 | *GPBP1* |
| 166 | 16 | 25300001 | 25490000 | 0.190 | 1 | *NDUFS4* |
| 167 | 16 | 42140001 | 42330000 | 0.190 | 1 | *CDH6* |
| 168 | 16 | 53590001 | 53780000 | 0.190 | 1 | *CDH18* |
| 169 | 17 | 5200001 | 5390000 | 0.190 | 1 | *LOC101108423* |
| 170 | 17 | 41480001 | 41670000 | 0.190 | 1 | *GRIA2* |
| 171 | 17 | 50230001 | 50420000 | 0.190 | 1 | *AACS* |
| 172 | 17 | 55560001 | 55750000 | 0.190 | 2 | *CCDC60, HSPB8* |
| 173 | 17 | 63290001 | 63480000 | 0.190 | 3 | *UBE3B, MYO1H, FOXN4* |
| 174 | 17 | 63780001 | 64000000 | 0.220 | 3 | *DAO, SSH1, CORO1C* |
| 175 | 17 | 64830001 | 65020000 | 0.190 | 3 | *KIAA1671, CRYBB3, CRYBB2* |
| 176 | 17 | 67910001 | 68100000 | 0.190 | 4 | *C17H22orf31, KREMEN1, RHBDD3, EWSR1* |
| 177 | 17 | 68450001 | 68730000 | 0.280 | 3 | *ASCC2, MTMR3, HORMAD2* |
| 178 | 18 | 4450001 | 4640000 | 0.190 | 1 | *LRRK1* |
| 179 | 18 | 16100001 | 16290000 | 0.190 | 1 | *AGBL1* |
| 180 | 18 | 19500001 | 19700000 | 0.200 | 3 | *LRRK1, AGBL1, ACAN* |
| 181 | 18 | 20220001 | 20410000 | 0.190 | 3 | *WDR93, ANPEP, LOC101105107* |
| 182 | 18 | 21900001 | 22090000 | 0.190 | 3 | *PDE8A, RPS17, CPEB1* |
| 183 | 18 | 23520001 | 23880000 | 0.360 | 1 | *EFL1* |
| 184 | 18 | 33990001 | 34180000 | 0.190 | 1 | *STXBP6* |
| 185 | 18 | 35420001 | 35610000 | 0.190 | 1 | *NOVA1* |
| 186 | 18 | 39130001 | 39320000 | 0.190 | 1 | *PRKD1* |
| 187 | 18 | 56090001 | 56280000 | 0.190 | 1 | *SLC24A4, RINA3* |
| 188 | 18 | 58840001 | 59030000 | 0.190 | 1 | *SYNE3* |
| 189 | 19 | 1700001 | 1890000 | 0.190 | 1 | *SLC4A7* |
| 190 | 19 | 9910001 | 10100000 | 0.190 | 1 | *STAC* |
| 191 | 19 | 10540001 | 10730000 | 0.190 | 2 | *TRANK1, DCLK3, GOLGA4* |
| 192 | 19 | 11820001 | 12010000 | 0.190 | 2 | *EXOG, SCN5A* |
| 193 | 19 | 14000001 | 14250000 | 0.250 | 1 | *ULK4* |
| 194 | 19 | 15910001 | 16100000 | 0.190 | 2 | *TOPAZ1, TCAIM* |
| 195 | 19 | 16130001 | 16340000 | 0.210 | 3 | *ZNF445, ZNF852, LOC101120528* |
| 196 | 19 | 17720001 | 17910000 | 0.190 | 1 | *LMCD1* |
| 197 | 19 | 32390001 | 32580000 | 0.190 | 5 | *LMOD3, ARL6IP5, UBA3, TMF1, EOGT* |
| 198 | 19 | 34010001 | 34310000 | 0.300 | 1 | *SUCLG2* |
| 199 | 19 | 36410001 | 36600000 | 0.190 | 1 | *CACNA2D3* |
| 200 | 19 | 51550001 | 51740000 | 0.190 | 4 | *CATHL3, BAC5, SC5, CDC25A* |
| 201 | 19 | 54050001 | 54240000 | 0.190 | 3 | *CLEC3B, ZDHHC3, SEC13* |
| 202 | 19 | 56620001 | 56810000 | 0.190 | 2 | *PPARG, SYN2* |
| 203 | 19 | 58830001 | 59020000 | 0.190 | 5 | *LOC101106975, CNBP, COPG1, LOC101106288, RAB7A* |
| 204 | 20 | 10320001 | 10510000 | 0.190 | 4 | *PNPLA1, C20H6orf222, PXT1, KCTD20* |
| 205 | 20 | 10810001 | 11000000 | 0.190 | 4 | *CPNE5, PPIL1, C20H6orf89, PI16* |
| 206 | 20 | 13640001 | 13900000 | 0.260 | 3 | *KIF6, DAAM2, MOCS1* |
| 207 | 20 | 27320001 | 27530000 | 0.210 | 8 | *IER3, FLOT1, TUBB, MDC1, NRM, PPP1R18, DHX16, ATAT1* |
| 208 | 20 | 46060001 | 46250000 | 0.190 | 1 | *SLC35B3* |
| 209 | 21 | 4510001 | 4720000 | 0.210 | 2 | *CHORDC1, NAALAD2* |
| 210 | 21 | 8810001 | 9000000 | 0.190 | 1 | *PICALM* |
| 211 | 21 | 16700001 | 16890000 | 0.190 | 3 | *PICALM, TENM4, NARS2* |
| 212 | 21 | 17520001 | 17740000 | 0.220 | 3 | *INTS4, AAMDC, RSF1* |
| 213 | 21 | 27270001 | 27460000 | 0.190 | 6 | *TBRG1, PANX3, SIAE, SPA17, VSIG2, ESAM* |
| 214 | 21 | 37600001 | 37790000 | 0.190 | 2 | *LOC101102598, LOC101104283* |
| 215 | 21 | 38480001 | 38690000 | 0.210 | 2 | *LOC101106045, LOC101104869* |
| 216 | 21 | 40520001 | 40710000 | 0.190 | 5 | *STX5, WDR74, SLC3A2, LOC101112776, SLC22A8* |
| 217 | 21 | 41200001 | 41390000 | 0.190 | 2 | *LOC101120283, ATL3* |
| 218 | 21 | 42870001 | 43060000 | 0.190 | 2 | *SLC25A45, SCYL1* |
| 219 | 22 | 14670001 | 14860000 | 0.190 | 1 | *LGI1* |
| 220 | 22 | 22870001 | 23060000 | 0.190 | 2 | *NT5C2, INA* |
| 221 | 22 | 42410001 | 42600000 | 0.190 | 1 | *CPXM2* |
| 222 | 23 | 2040001 | 2230000 | 0.190 | 2 | *GALR1, ZNF24* |
| 223 | 23 | 2950001 | 3140000 | 0.190 | 2 | *KCTD1, SS18* |
| 224 | 23 | 40200001 | 40390000 | 0.190 | 1 | *LAMA1, ARHGAP28* |
| 225 | 24 | 3920001 | 4110000 | 0.190 | 5 | *GLYR1, UBN1, PPL, NAGPA, C24H16orf89* |
| 226 | 24 | 16380001 | 16570000 | 0.190 | 3 | *RPS15A, SMG1, LOC101115593* |
| 227 | 24 | 21010001 | 21200000 | 0.190 | 2 | *COG7, GGA2* |
| 228 | 24 | 22660001 | 22850000 | 0.190 | 3 | *LCMT1, AQP8, ZKSCAN2* |
| 229 | 24 | 37580001 | 37770000 | 0.190 | 3 | *BAIAP2L1, TECPR1, LMTK2* |
| 230 | 25 | 19550001 | 19740000 | 0.190 | 1 | *REEP3* |
| 231 | 25 | 41320001 | 41510000 | 0.190 | 3 | *FAM35A, LOC101115343, LOC101113823* |
| 232 | 25 | 42950001 | 43140000 | 0.190 | 1 | *VSTM4, FAM170B, C25H10orf71* |
| 233 | 26 | 6000001 | 6410000 | 0.410 | 1 | *WDR17, GPM6A* |
| 234 | 26 | 29860001 | 30050000 | 0.190 | 1 | *UNC5D* |
| 235 | 26 | 40130001 | 40320000 | 0.190 | 1 | *THRB* |
| 236 | 27 | 310001 | 500000 | 0.190 | 2 | *LOC105605310, CRLF2* |
| 237 | 27 | 4590001 | 4780000 | 0.190 | 1 | *STS* |
| 238 | 27 | 26790001 | 27030000 | 0.240 | 1 | *IL1RAPL1* |
| 239 | 27 | 80220001 | 80410000 | 0.190 | 2 | *MAMLD1, MTM1* |
| 240 | 27 | 117440001 | 117630000 | 0.190 | 2 | *PAK3, CAPN6* |
| 241 | 27 | 125530001 | 125720000 | 0.190 | 7 | *TAF7L, TIMM8A, BTK, RPL36A, GLA, HNRNPH2, ARMCX4* |
| 242 | 27 | 126730001 | 126920000 | 0.190 | 1 | *TNMD* |

**Supplementary Tables S4.** The candidate regions spanning genes within the HR (HR vs LR) sheep group identified *via* F_ST_.

| **Reg.** | **Chr.** | **Start** | **Stop** | **Size (Mb)** | **No of**  **Genes** | **Genes** |
| --- | --- | --- | --- | --- | --- | --- |
| 1 | 1 | 175400001 | 175540000 | 0.140 | 2 | *SLC9C1, CD200* |
| 2 | 1 | 227000001 | 227110000 | 0.110 | 2 | *MLF1, RSRC1* |
| 3 | 1 | 253450001 | 253580000 | 0.130 | 2 | *SLCO2A1, RAB6B* |
| 4 | 2 | 2470001 | 2620000 | 0.150 | 3 | *C5, TRAF1, PHF19* |
| 5 | 2 | 51980001 | 52100000 | 0.120 | 4 | *GNE, CLTA, CCIN, GLIPR2* |
| 6 | 2 | 53110001 | 53250000 | 0.140 | 4 | *DNAJB5, LOC101110212, C2H9orf131, KIAA1045* |
| 7 | 2 | 60990001 | 61150000 | 0.160 | 1 | *LOC101121827* |
| 8 | 2 | 126600001 | 126750000 | 0.150 | 2 | *PPP1R1C, SSFA2* |
| 9 | 2 | 129330001 | 129470000 | 0.140 | 2 | *ZNF385B, LOC105608948* |
| 10 | 2 | 146510001 | 146680000 | 0.170 | 3 | *FAP, GCG, DPP4* |
| 11 | 2 | 201150001 | 201340000 | 0.190 | 4 | *LOC101102297, LOC105610167, TYW5, C2H2orf69* |
| 12 | 3 | 32270001 | 32400000 | 0.130 | 2 | *DNAJC27, EFR3B* |
| 13 | 3 | 45890001 | 45990000 | 0.100 | 1 | *COMMD1* |
| 14 | 3 | 96170001 | 96290000 | 0.120 | 13 | *DCTN1, LOC101116640, C3H2orf81, WDR54, RTKN, INO80B, WBP1, MOGS, MRPL53, CCDC142, TTC31, LBX2, LOC105607909* |
| 15 | 3 | 172510001 | 172660000 | 0.150 | 3 | *STAB2, LOC101123368, NT5DC3* |
| 16 | 3 | 183070001 | 183220000 | 0.150 | 1 | *DENND5B* |
| 17 | 3 | 194020001 | 194180000 | 0.160 | 1 | *PDE3A* |
| 18 | 3 | 195030001 | 195140000 | 0.110 | 1 | *LOC105611562* |
| 19 | 3 | 202380001 | 202540000 | 0.160 | 3 | *LRP6, LOC105611787, LOC105614841* |
| 20 | 3 | 213480001 | 213580000 | 0.100 | 6 | *SH3BP1, PDXP, LGALS1, NOL12, TRIOBP, TRNAG-CCC* |
| 21 | 3 | 224050001 | 224250000 | 0.200 | 10 | *KLHDC7B, SYCE3, CPT1B, CHKB, MAPK8IP2, ARSA, SHANK3, ACR, LOC105606338, RABL2B* |
| 22 | 4 | 48760001 | 48940000 | 0.180 | 3 | *SLC26A3, LOC105611149, DLD* |
| 23 | 4 | 92040001 | 92210000 | 0.170 | 1 | *SND1* |
| 24 | 5 | 105850001 | 105950000 | 0.100 | 1 | *MAN2A1(Forwards)* |
| 25 | 6 | 5590001 | 5780000 | 0.190 | 1 | *MAD2L1* |
| 26 | 6 | 24810001 | 24940000 | 0.130 | 4 | *DNAJB14, DAPP1, H2AFZ, LAMTOR3* |
| 27 | 6 | 36180001 | 36330000 | 0.150 | 4 | *PYURF, HERC6, PIGY, HERC5* |
| 28 | 6 | 53520001 | 53620000 | 0.100 | 1 | *LOC101109539* |
| 29 | 6 | 62460001 | 62560000 | 0.100 | 1 | *LOC101111286* |
| 30 | 6 | 110620001 | 110820000 | 0.200 | 1 | \| *PROM1* \| \| --- \| \|  \| |
| 31 | 7 | 16570001 | 16710000 | 0.140 | 1 | *LOC105611453* |
| 32 | 8 | 27830001 | 27930000 | 0.100 | 4 | *PPIL6, CD164, LOC101108397, POLE4* |
| 33 | 8 | 80110001 | 80410000 | 0.300 | 1 | *ARID1B* |
| 34 | 8 | 89570001 | 89670000 | 0.100 | 2 | *SMOC2 (Backwards) THBS2 (Forwards)* |
| 35 | 9 | 13550001 | 13710000 | 0.160 | 11 | *SCRT1, DGAT1, HSF1, BOP1, MROH1, HGH1, MAF1, SHARPIN, LOC101113599, GPAA1, EXOSC4* |
| 36 | 9 | 32520001 | 32630000 | 0.110 | 2 | *LOC105609197, TRNAR-CCU* |
| 37 | 9 | 33870001 | 34000000 | 0.130 | 4 | *PCMTD1, ST18, LOC105616019, LOC105616020* |
| 38 | 9 | 43280001 | 43420000 | 0.140 | 3 | *DNAJC5B, CRH, TRIM55* |
| 39 | 9 | 66700001 | 66830000 | 0.130 | 1 | *LOC105613281* |
| 40 | 10 | 32510001 | 32680000 | 0.170 | 2 | *LOC101113604, LNX2* |
| 41 | 11 | 9330001 | 9470000 | 0.140 | 1 | *PPM1E* |
| 42 | 12 | 29840001 | 30020000 | 0.180 | 1 | *KIF26B, LOC105616515* |
| 43 | 12 | 39860001 | 39960000 | 0.100 | 3 | *PLOD1, NPPB, KIAA2013* |
| 44 | 12 | 54340001 | 54490000 | 0.150 | 4 | *TNN, KIAA0040, LOC105612225, LOC105612227* |
| 45 | 12 | 78550001 | 79050000 | 0.500 | 14 | *CSRP1, LOC105616602, TNNI1, LAD1, TNNT2, IPO9, SHISA4, LMOD1, LOC105610030, LOC105610031, TIMM17A, RNPEP, ELF3, LOC105610032* |
| 46 | 13 | 37980001 | 38160000 | 0.180 | 1 | *SLC24A3* |
| 47 | 13 | 50610001 | 50720000 | 0.110 | 4 | *PANK2, MAVS, AP5S1, LOC105606256* |
| 48 | 14 | 48360001 | 48470000 | 0.110 | 2 | *EID2, LOC101116592* |
| 49 | 14 | 53230001 | 53330000 | 0.100 | 3 | *SAE1, LOC105616907, BBC3* |
| 50 | 15 | 5560001 | 5680000 | 0.120 | 1 | *MMP20* |
| 51 | 15 | 17590001 | 17690000 | 0.100 | 2 | *C15H11orf65, LOC101105356* |
| 52 | 15 | 20560001 | 20730000 | 0.170 | 1 | *LOC105602151* |
| 53 | 16 | 1420001 | 1520000 | 0.100 | 2 | *LOC105602434, SPDL1* |
| 54 | 16 | 2310001 | 2420000 | 0.110 | 3 | *KCNIP1, LOC105602444, KCNMB1* |
| 55 | 16 | 53590001 | 53800000 | 0.210 | 2 | *CDH18, TRNAW-CCA* |
| 56 | 17 | 10830001 | 10980000 | 0.150 | 1 | *LOC105602723, LOC105602722* |
| 57 | 17 | 51260001 | 51380000 | 0.120 | 3 | *LOC105602862, CCDC92, DNAH10* |
| 58 | 18 | 23690001 | 23940000 | 0.250 | 1 | *MEX3B (Backwards)* |
| 59 | 18 | 32210001 | 32470000 | 0.260 | 10 | *PTPN9, SIN3A, MAN2C1, NEIL1, COMMD4, LOC105603152, LOC105603296, LOC105603153, C18H15orf39, LOC105603154* |
| 60 | 19 | 910001 | 1070000 | 0.160 | 2 | *LOC105603325, LOC105603324* |
| 61 | 19 | 9950001 | 10090000 | 0.140 | 1 | *STAC* |
| 62 | 19 | 17010001 | 17120000 | 0.110 | 3 | *SETD5, LHFPL4, LOC105603622* |
| 63 | 21 | 38480001 | 38690000 | 0.210 | 7 | *LOC105601927, LOC101106045, LOC101104869, LOC101106295, LOC105604093, LOC101106556, LOC101106809* |
| 64 | 21 | 40100001 | 40250000 | 0.150 | 5 | *SCGB2A2, ASRGL1, AHNAK, SCGB1A1, LOC105604099* |
| 65 | 21 | 40530001 | 40650000 | 0.120 | 5 | *SLC3A2, LOC101112776, STX5, WDR74, CHRM1* |
| 66 | 22 | 45680001 | 45850000 | 0.170 | 1 | *DOCK1* |
| 67 | 24 | 36540001 | 36640000 | 0.100 | 2 | *LOC101110202, LOC101102118* |
| 68 | 24 | 41370001 | 41560000 | 0.190 | 6 | *GPR146, C24H7orf50, LOC105604882, LOC101120042, ADAP1, GET4* |
| 69 | 25 | 19580001 | 19720000 | 0.140 | 2 | *REEP3, LOC101122571* |
| 70 | 27 | 7320001 | 7550000 | 0.230 | 1 | *LOC101120895* |
| 71 | 27 | 7910001 | 8080000 | 0.170 | 1 | *MID1* |
| 72 | 27 | 62420001 | 62520000 | 0.100 | 2 | *CHIC1, LOC105605506* |
| 73 | 27 | 62870001 | 63020000 | 0.150 | 5 | *LOC101112542, MIR374A, ZCCHC13, LOC101103953, TRNAW-CCA* |
| 74 | 27 | 65110001 | 65220000 | 0.110 | 1 | *LOC101105221* |
| 75 | 27 | 77600001 | 77860000 | 0.260 | 13 | *PDZD4, SSR4, IDH3G, SRPK3, PLXNB3, ABCD1, BCAP31, SLC6A8, LOC105605544, PNCK, DUSP9, FAM58A, ATP2B3* |
| 76 | 27 | 79090001 | 79270000 | 0.180 | 1 | *CNGA2* |
| 77 | 27 | 98930001 | 99060000 | 0.130 | 4 | *LOC105605718, LOC105605587, TMEM255A, ZBTB33* |
| 78 | 27 | 102240001 | 102410000 | 0.170 | 2 | *XIAP, STAG2, LOC105605596* |
| 79 | 27 | 110390001 | 110550000 | 0.160 | 2 | *AGTR2 (Backwards), DOCK11 (Forwards)* |

**Supplementary Table S5** The candidate regions spanning genes within the HR (HR vs LR) sheep group identified *via* XP-EHH.

| **Reg.** | **Chr.** | **Start** | **Stop** | **Size (Mb)** | **No of**  **Genes** | **Genes** |
| --- | --- | --- | --- | --- | --- | --- |
| 1 | 1 | 67320001 | 67510000 | 0.190 | 2 | *BARHL2, ZNF644* |
| 2 | 1 | 102630001 | 102890000 | 0.260 | 10 | *S100A1, CHTOP, SNAPIN, ILF2, TRNAM-CAU, NPR1, INTS3, SLC27A3, GATAD2B, DENND4B* |
| 3 | 1 | 115740001 | 116440000 | 0.700 | 1 | *FAM78B* |
| 4 | 1 | 122490001 | 122680000 | 0.190 | 1 | *TIAM1* |
| 5 | 1 | 227850001 | 228160000 | 0.310 | 1 | *VEPH1* |
| 6 | 1 | 253250001 | 253440000 | 0.190 | 2 | *RYK, SLCO2A1* |
| 7 | 2 | 53060001 | 53500000 | 0.440 | 6 | *FAM214B, STOML2, PIGO, C2H9orf131, DNAJB5, KIAA1045* |
| 8 | 2 | 60250001 | 60450000 | 0.200 | 1 | *PCSK5* |
| 9 | 2 | 62940001 | 63160000 | 0.220 | 1 | *ANXA1* |
| 10 | 2 | 67670001 | 67860000 | 0.190 | 2 | *TMEM252, PGM5* |
| 11 | 2 | 84730001 | 84930000 | 0.200 | 1 | *BNC2* |
| 12 | 2 | 85810001 | 86000000 | 0.190 | 1 | *ADAMTSL1* |
| 13 | 2 | 173240001 | 173430000 | 0.190 | 1 | *CXCR4* |
| 14 | 2 | 201190001 | 201380000 | 0.190 | 2 | *TYW5, C2H2orf69,* |
| 15 | 2 | 205840001 | 206040000 | 0.200 | 1 | *PARD3B* |
| 16 | 2 | 211410001 | 211640000 | 0.230 | 1 | *TRNAC-GCA,* |
| 17 | 3 | 1280001 | 1470000 | 0.190 | 1 | *COL5A1* |
| 18 | 3 | 23660001 | 23900000 | 0.240 | 2 | *LOC101105564, DDX1* |
| 19 | 3 | 65880001 | 66110000 | 0.230 | 2 | *VRK2, LOC101111446* |
| 20 | 3 | 94680001 | 94970000 | 0.290 | 3 | *EXOC6B, EMX1, SPR* |
| 21 | 3 | 97640001 | 97930000 | 0.290 | 1 | *LOC105612339* |
| 22 | 3 | 114790001 | 115060000 | 0.270 | 1 | *SYT1* |
| 23 | 3 | 136000001 | 136190000 | 0.190 | 10 | *CERS5, GPD1, SMARCD1, ASIC1, LOC105612625, RACGAP1, LOC105614724, AQP6, AQP5, AQP2* |
| 24 | 4 | 42410001 | 42630000 | 0.220 | 1 | *MAGI2* |
| 25 | 4 | 91770001 | 91960000 | 0.190 | 5 | *GCC1, ARF5, FSCN3, PAX4, SND1* |
| 26 | 4 | 92290001 | 92480000 | 0.190 | 2 | *SND1, LRRC4* |
| 27 | 5 | 19550001 | 19800000 | 0.250 | 6 | *LOC105615229, SLC22A5, LOC105611480, SLC22A4, P4HA2, PDLIM4* |
| 28 | 5 | 44110001 | 44320000 | 0.210 | 2 | *LOC105607063, LOC101104574* |
| 29 | 5 | 99170001 | 99360000 | 0.190 | 2 | *PAM, GIN1* |
| 30 | 6 | 70710001 | 70900000 | 0.190 | 2 | *SRD5A3, TMEM165* |
| 31 | 7 | 12240001 | 12430000 | 0.190 | 4 | *VWA9, HACD3, SLC24A1, DENND4A* |
| 32 | 7 | 59600001 | 59820000 | 0.220 | 1 | *SEMA6D* |
| 33 | 7 | 86060001 | 86270000 | 0.210 | 1 | *LOC101111637* |
| 34 | 8 | 2430001 | 2620000 | 0.190 | 2 | *SENP6, MYO6* |
| 35 | 8 | 27770001 | 28010000 | 0.240 | 8 | *AK9, ZBTB24, MICAL1, SMPD2, PPIL6, POLE4, CD164, LOC101108397* |
| 36 | 8 | 42360001 | 42550000 | 0.190 | 1 | *LOC101107027* |
| 37 | 8 | 63150001 | 63500000 | 0.350 | 3 | *NHSL1, ARFGEF3, HEBP2* |
| 38 | 9 | 27500001 | 27730000 | 0.230 | 2 | *TRIB1, NSMCE2,* |
| 39 | 9 | 59490001 | 59690000 | 0.200 | 1 | *SLITRK1* |
| 40 | 10 | 25910001 | 26100000 | 0.190 | 2 | *DCLK1, NBEA* |
| 41 | 10 | 41140001 | 41350000 | 0.210 | 1 | *LOC101121273* |
| 42 | 11 | 3220001 | 3410000 | 0.190 | 1 | \| *LOC101119575* \| \| --- \| \|  \| |
| 43 | 11 | 6640001 | 6830000 | 0.190 | 1 | *ANKFN1* |
| 44 | 11 | 10170001 | 10360000 | 0.190 | 3 | *CLTC, VMP1, PTRH2* |
| 45 | 11 | 44950001 | 45160000 | 0.210 | 2 | *NSF, LOC101120590* |
| 46 | 12 | 43180001 | 43380000 | 0.200 | 1 | *SLC45A1* |
| 47 | 12 | 62290001 | 62480000 | 0.190 | 2 | *NMNAT2, LAMC2* |
| 48 | 13 | 38000001 | 38210000 | 0.210 | 1 | *SLC24A3* |
| 49 | 15 | 1420001 | 1610000 | 0.190 | 1 | *GRIA4* |
| 50 | 15 | 17190001 | 17610000 | 0.420 | 5 | *CUL5, ACAT1, ATM, C15H11orf65, NPAT* |
| 51 | 15 | 19640001 | 19850000 | 0.210 | 2 | *ZC3H12C, RDX* |
| 52 | 15 | 20580001 | 20780000 | 0.200 | 1 | *LOC105602151* |
| 53 | 15 | 46800001 | 46990000 | 0.190 | 11 | *LOC101122721, LOC101122976, LOC101117874, LOC101118131, LOC101123233, LOC101123492, LOC101101917, LOC101102173, LOC101102419, LOC101118638, LOC101118897* |
| 54 | 16 | 12310001 | 12510000 | 0.200 | 3 | *LOC105602490, MAST4, CD180* |
| 55 | 16 | 16120001 | 16310000 | 0.190 | _ | *_* |
| 56 | 16 | 24220001 | 24430000 | 0.210 | 3 | *LOC101113531, LOC101118133, LOC105602535* |
| 57 | 17 | 13120001 | 13350000 | 0.230 | 3 | *HHIP, LOC105602737, LOC105602739* |
| 58 | 17 | 43980001 | 44170000 | 0.190 | 6 | *CHFR, GOLGA3, ANKLE2, PGAM5, PXMP2, P2RX2* |
| 59 | 17 | 50680001 | 50870000 | 0.190 | 2 | *LOC105602859, NCOR2* |
| 60 | 17 | 52590001 | 52790000 | 0.200 | 7 | *CLIP1, LOC105602870, VPS33A, LOC105602871, DIABLO, B3GNT4, LRRC43* |
| 61 | 17 | 56280001 | 56490000 | 0.210 | 2 | *TAOK3, SUDS3* |
| 62 | 17 | 59120001 | 59310000 | 0.190 | 1 | *LOC105602900* |
| 63 | 18 | 33800001 | 34000000 | 0.200 | 1 | *STXBP6* |
| 64 | 18 | 41810001 | 42060000 | 0.250 | 1 | *AKAP6* |
| 65 | 19 | 12390001 | 12580000 | 0.190 | 9 | *TTC21A, CSRNP1, LOC105603376, LOC105603377, XIRP1, LOC105603378, CX3CR1, CCR8, SLC25A38* |
| 66 | 22 | 46350001 | 46540000 | 0.190 | 2 | *PTPRE, MKI67* |
| 67 | 23 | 26380001 | 26570000 | 0.190 | 1 | *DSC3* |
| 68 | 24 | 32560001 | 32760000 | 0.200 | 7 | *GATSL2, WBSCR16, LOC105604768, LOC101112784, TRNAC-ACA, NCF1, GTF2I* |
| 69 | 25 | 5610001 | 5800000 | 0.190 | 1 | *MAP10* |
| 70 | 27 | 83660001 | 83870000 | 0.210 | _ | *_* |
| 71 | 27 | 100310001 | 100500000 | 0.190 | 1 | *LOC105605719* |
| 72 | 27 | 103540001 | 103750000 | 0.210 | 1 | *TENM1* |
| 73 | 27 | 105090001 | 105280000 | 0.190 | 1 | *PRR32* |
| 74 | 27 | 106830001 | 107040000 | 0.210 | 1 | *LOC105605601* |
| 75 | 27 | 116660001 | 116850000 | 0.190 | 2 | *TRPC5, TRPC5OS* |
| 76 | 27 | 134120001 | 134330000 | 0.210 | 1 | *PCDH11X* |

**Supplementary Table S6** Candidate regions that are specific to the high resistance (HR) cohort as identified by ROH analysis

| **Region** | **OAR** | **Start** | **Stop** | **Size (Mb)** | **No. of**  **SNPs** | **No. of genes** | **Genes** |
| --- | --- | --- | --- | --- | --- | --- | --- |
| 1 | 1 | 102960696 | 102986833 | 0.026 | 10 | 2 | *NUP210L, LOC105608943* |
| 2 | 2 | 39326712 | 39364679 | 0.038 | 3 | 1 | *EBF2* |
| 3 | 2 | 67955833 | 68075151 | 0.119 | 22 | 4 | *LOC105611725, LOC101116383, LOC105607922, LOC101108887* |
| 4 | 2 | 122505774 | 122512143 | 0.006 | 4 | 1 | *FSIP2* |
| 5 | 3 | 105188610 | 105221230 | 0.033 | 7 | 2 | *ACOXL, BCL2L11* |
| 6 | 3 | 129702880 | 129753390 | 0.051 | 10 | 1 | *SOCS2* |
| 7 | 3 | 183129985 | 183130932 | 0.001 | 2 | 1 | *TRNAC-GCA* |
| 8 | 3 | 183135436 | 183234842 | 0.099 | 16 | 1 | *DENND5B* |
| 9 | 4 | 35647908 | 35793980 | 0.146 | 1 | 1 | *SEMA3D* |
| 10 | 4 | 69220420 | 69224698 | 0.004 | 2 | 1 | *SKAP2* |
| 11 | 4 | 94343234 | 94425969 | 0.083 | 11 | 1 | *COPG2* |
| 12 | 6 | 24689427 | 24691924 | 0.002 | 2 | 1 | *TRNAC-GCA* |
| 13 | 6 | 116809590 | 116996609 | 0.187 | 19 | 5 | *PCGF3, MFSD7, ATP5I, PDE6B, PIGG* |
| 14 | 7 | 32959471 | 32960674 | 0.001 | 2 | 1 | *INAFM2* |
| 15 | 9 | 60265603 | 60265603 | 0.000 | 1 | 1 | *RAD21* |
| 16 | 10 | 30643970 | 30721249 | 0.077 | 20 | 1 | *TRNAW-CCA* |
| 17 | 10 | 36197592 | 36369348 | 0.172 | 20 | 4 | *GJB6, GJB2, GJA3, ZMYM2* |
| 18 | 11 | 12276044 | 12334873 | 0.059 | 12 | 1 | *USP32* |
| 19 | 11 | 26580667 | 26731105 | 0.150 | 24 | 18 | *PHF23, GABARAP, CTDNEP1, ELP5, CLDN7, SLC2A4, YBX2, EIF5A, GPS2, NEURL4, ACAP1, KCTD11, TMEM95, TNK1, PLSCR3, TMEM256, NLGN2, SPEM1* |
| 20 | 13 | 17132098 | 17218518 | 0.086 | 21 | 1 | *CCNY* |
| 21 | 13 | 53241515 | 53308183 | 0.067 | 10 | 2 | *TPD52L2, ABHD16B* |
| 22 | 15 | 958579 | 1427669 | 0.469 | 61 | 5 | *MRE11A, ANKRD49, AASDHPPT, KBTBD3, MSANTD4* |
| 23 | 15 | 47425386 | 47536716 | 0.111 | 23 | 5 | *LOC101101918, LOC101104683, LOC101102175, LOC101104931, LOC101102421,* |
| 24 | 15 | 67884311 | 67990507 | 0.106 | 23 | 1 | *LOC101115157* |
| 25 | 16 | 71407458 | 71455062 | 0.048 | 11 | 1 | *MRPL36* |
| 26 | 18 | 23665103 | 23826989 | 0.162 | 25 | 1 | *MEX3B* |
| 27 | 20 | 22272855 | 22366433 | 0.094 | 15 | 3 | *PGK2, LOC101118060, CRISP1* |
| 28 | 21 | 38646061 | 38906932 | 0.261 | 21 | 6 | *LOC101106556, LOC101106809, LOC101105115, LOC101107054, PAG6, LOC105604095,* |
| 29 | 27 | 41443596 | 41451579 | 0.008 | 3 | 1 | *EFHC2* |
| 30 | 27 | 47955566 | 47955566 | 0.000 | 1 | 1 | *PFKFB1* |

**Supplementary Table S7** The top most Enriched functional clusters and their enrichment scores following DAVID analysis for genes identified by all methodologies and that were specific to HR Tunisian sheep group.

| ***Category*** | ***ID*** | ***Term*** | ***Gene Count*** | ***P-value*** | ***Genes*** | ***Benjamini*** |
| --- | --- | --- | --- | --- | --- | --- |
| KEGG | oas04142 | Lysosome | 4 | 0.0018 | *GGA1, CLTA, ARSA, CD164* | 0.1967 |
| KEGG | oas05206 | MicroRNAs in cancer | 4 | 0.0087 | *RECK, ATM, BCL2L11, TNN* | 0.3645 |
| GOTERM_BP | GO:0007339 | binding of sperm to zona pellucida | 3 | 0.0094 | *ACR, CRISP1, ARSA* | 0.9519 |
| KEGG | oas03440 | Homologous recombination | 2 | 0.0139 | *MRE11, ATM* | 0.3645 |
| KEGG | oas05166 | Human T-cell leukemia virus 1 infection | 4 | 0.0178 | *MMP7, GPS2, ATM, MAD2L1* | 0.3645 |
| KEGG | oas04068 | FoxO signaling pathway | 3 | 0.0188 | *ATM, BCL2L11, GABARAP* | 0.3645 |
| KEGG | oas00600 | Sphingolipid metabolism | 2 | 0.0203 | *ARSA, SMPD2* | 0.3645 |
| GOTERM_MF | GO:0008270 | zinc ion binding | 12 | 0.0248 | *MEX3B, MMP7, ZMYM2, MMP27, MAN2A1, MICAL1, PHF23, RNF38, NSMCE2, CBLL1, ST18, MMP20* | 0.8955 |
| KEGG | oas04210 | Apoptosis | 3 | 0.0248 | *TUBA8, ATM, BCL2L11* | 0.3830 |
| GOTERM_BP | GO:0007369 | gastrulation | 2 | 0.0347 | *EXT1, DLD* | 1.0000 |
| KEGG | oas00010 | Glycolysis / Gluconeogenesis | 2 | 0.0351 | *PGK2, DLD* | 0.4011 |
| KEGG | oas04530 | Tight junction | 3 | 0.0371 | *TIAM1, TUBA8, CLDN7* | 0.4011 |
| GOTERM_MF | GO:0005198 | structural molecule activity | 4 | 0.0492 | *ANXA1, CLDN7, CLTA, COPG2* | 0.9893 |

**Supplementary Table S8** Candidate regions associated genes overlapped between F_ST_ and XP-EHH within different comparisons among study sheep groups.

| **Group** | **Reg** | **OAR** | **Start** | **Stop** | **Size (Mb)** | **Method** | | **Comparison** | **No. of genes** | **Genes (Top gene**)** |
| --- | --- | --- | --- | --- | --- | --- | --- | --- | --- | --- |
| **G1** | 1 | 2 | 121860001 | 122090000 | 0.230 | XP-EHH | F_ST_ | G1vsG4 | 1 | *LOC105608882* |
|  | 2 | 3 | 153770001 | 153890000 | 0.120 | _ | F_ST_ | G1vsG2; G1vsG4 | 1 | *LOC105609946* |
|  | 3 | 13 | 48470001 | 48870000 | 0.400 | XP-EHH | _ | G1vsG3, G1vsG4 | 1 | *BMP2* |
|  | 4 | 13 | 49050001 | 49380000 | 0.330 | XP-EHH | _ | G1vsG3; G1vsG4 | 1 | *LOC101118207, LOC101110166* |
|  | 5 | 13 | 49110001 | 50130000 | 1.020 | XP-EHH | _ | G1vsG3; G1vsG4 | 1 | *HAO1* |
|  | 6 | 15 | 740001 | 860000 | 0.120 | XP-EHH | F_ST_ | G1vsG4 | 1 | *IZUMO1R* |
|  | 7 | 15 | 3550001 | 3770000 | 0.220 | XP-EHH | F_ST_ | G1vsG4 | 1 | *LOC101117272* |
|  | 8 | 18 | 32270001 | 32450000 | 0.180 | _ | F_ST_ | G1vsG2; G1vsG4 | 6 | *PTPN9, SIN3A, MAN2C1, NEIL1, COMMD4, C18H15orf39* |
|  | 9 | 27 | 9630001 | 9850000 | 0.220 | _ | F_ST_ | G1vsG2; G1vsG3 | 1 | *FRMPD4* |
|  | 10 | 27 | 109860001 | 110080000 | 0.220 | _ | F_ST_ | G1vsG3; G1vsG4 | 2 | *ZCCHC12, IL13RA1* |
| **G2** | 1 | 1 | 23830001 | 24030000 | 0.200 | _ | F_ST_ | G2vsG3; G2vsG4 | 1 | *AGBL4* |
|  | 2 | 2 | 243590001 | 243800000 | 0.210 | _ | F_ST_ | G2vsG1; G2vsG4 | 3 | *WNT4, CDC42, HSPG2* |
|  | 3 | 4 | 68710001 | 68950000 | 0.240 | _ | F_ST_ | G2vsG3; G2vsG4 | 12 | *EVX1, HOXA13, HOXA11, HOXA10, HOXA9, HOXA7, HOXA3, HOXA6, HOXA5, HOXA4, HOXA2, HOXA1* |
|  | 4 | 8 | 34650001 | 34750000 | 0.100 | _ | F_ST_ | G2vsG1; G2vsG3 | 1 | *LOC105609885* |
|  | 5 | 10 | 35800001 | 36220000 | 0.420 | _ | F_ST_ | G2vsG3; G2vsG4 | 8 | *SKA3, SAP18, LATS2, XPO4, N6AMT2, IL17D, IFT88, CRYL1* |
|  | 6 | 12 | 9390001 | 9530000 | 0.140 | _ | F_ST_ | G2vsG1; G2vsG4 | 1 | *LOC105612678* |
|  | 7 | 12 | 78240001 | 78520000 | 0.280 | _ | F_ST_ | G2vsG1; G2vsG4; G2vsG3 | 2 | *UBE2T, LGR6* |
|  | 8 | 13 | 65560001 | 65700000 | 0.140 | XP-EHH | F_ST_ | G2vsG4 | 2 | *RBL1, MROH8* |
|  | 9 | 17 | 59480001 | 59670000 | 0.190 | _ | F_ST_ | G2vsG1; G2vsG3; G2vsG4 | 1 | *LOC105602900* |
|  | 10 | 20 | 20620001 | 20760000 | 0.140 | _ | F_ST_ | G2vsG1; G2vsG4 | 2 | *CD2AP, ADGRF2* |
|  | 11 | 20 | 40720001 | 41200000 | 0.480 | XP-EHH | _ | G2vsG1; G2vsG4 | 1 | *LOC105603854* |
|  | 12 | 20 | 38160001 | 38330000 | 0.170 | XP-EHH | F_ST_ | G2vsG1 | 3 | *RNF144B, DEK, KDM1B* |
|  | 13 | 20 | 40850001 | 41280000 | 0.430 | XP-EHH | F_ST_ | G2vsG1 | 1 | *LOC105603854* |
|  | 14 | 20 | 41700001 | 41880000 | 0.180 | XP-EHH | F_ST_ | G2vsG1 | 2 | *RNF182, MCUR1* |
|  | 15 | 24 | 19060001 | 19160000 | 0.100 | _ | F_ST_ | G2vsG1; G2vsG4 | 1 | *CRYM* |
|  | 16 | 25 | 18840001 | 19030000 | 0.190 | _ | F_ST_ | G2vsG1; G2vsG3 | 1 | *LOC101121824* |
|  | 17 | 27 | 9700001 | 9850000 | 0.150 | _ | F_ST_ | G2vsG1; G2vsG4 | 1 | *FRMPD4* |
|  | 18 | 27 | 110860001 | 110970000 | 0.110 | _ | F_ST_ | G2vsG3; G2vsG4 | 1 | *WDR44* |
| **G3** | 1 | 1 | 119540001 | 119900000 | 0.360 | _ | F_ST_ | G3vsG1; G3vsG4 | 4 | *MRPS6, SLC5A3, ATP5O, ITSN1* |
|  | 2 | 1 | 228640001 | 228790000 | 0.150 | _ | F_ST_ | G3vsG1; G3vsG2 | 1 | *LEKR1* |
|  | 3 | 1 | 63690001 | 63950000 | 0.260 | XP-EHH | _ | G3vsG1; G3vsG2 | 1 | *LMO4* |
|  | 4 | 2 | 111260001 | 111550000 | 0.290 | _ | F_ST_ | G3vsG2; G3vsG4 | 3 | *DDX60L, ANXA10, HIATL1* |
|  | 5 | 2 | 112440001 | 112770000 | 0.330 | _ | F_ST_ | G3vsG2; G3vsG4 | 1 | *OCA2, TRNAE-CUC* |
|  | 6 | 3 | 49160001 | 49310000 | 0.149 | _ | F_ST_ | G3vsG1; G3vsG4 | 1 | *LOC101121251* |
|  | 7 | 3 | 49160001 | 49310000 | 0.150 | _ | F_ST_ | G3vsG1; G3vsG4 | 1 | *LOC101121251* |
|  | 8 | 3 | 143390001 | 143530000 | 0.140 | _ | F_ST_ | G3vsG1; G3vsG4 | 1 | *ADAMTS20* |
|  | 9 | 5 | 78420001 | 78550000 | 0.130 | _ | F_ST_ | G3vsG1; G3vsG4 | 1 | *RASGRF2* |
|  | 10 | 18 | 56860001 | 57070000 | 0.210 | _ | F_ST_ | G3vsG1; G3vsG2 | 1 | *UNC79* |
|  | 11 | 20 | 1110001 | 1280000 | 0.170 | _ | F_ST_ | G3vsG2; G3vsG4 | 1 | *KHDRBS2* |
|  | 12 | 27 | 9630001 | 9850000 | 0.220 | _ | F_ST_ | G3vsG1; G3vsG4 | 1 | *FRMPD4* |
|  | 13 | 27 | 15930001 | 16130000 | 0.200 | _ | F_ST_ | G3vsG1; G3vsG4 | 2 | *CDKL5* |
|  | 14 | 27 | 78090001 | 78330000 | 0.240 | _ | F_ST_ | G3vsG1; G3vsG4 | 1 | *ZNF185, NSDHL* |
|  | 15 | 27 | 96650001 | 96820000 | 0.170 | _ | F_ST_ | G3vsG2; G3vsG4 | 2 | *HS6ST2* |
|  | 16 | 27 | 110860001 | 111050000 | 0.190 | _ | F_ST_ | G3vsG1, G3vsG2; G3vsG4 | 1 | *WDR44* |
|  | 17 | 27 | 117980001 | 118220000 | 0.240 | _ | F_ST_ | G3vsG1; G3vsG4 | 1 | *AMMECR1, CHRDL1, RGAG1* |
|  | 18 | 27 | 126970001 | 127250000 | 0.280 | _ | F_ST_ | G3vsG1; G3vsG2 | 1 | *PCDH19* |
| **G4** | 1 | 2 | 121830001 | 122090000 | 0.260 | _ | F_ST_ | G4vsG1; G4vsG2 | 1 | *LOC105608882* |
|  | 2 | 3 | 21290001 | 21430000 | 0.140 | _ | F_ST_ | G4vsG1; G4vsG2 | 1 | *LOC105607476* |
|  | 3 | 3 | 21310001 | 21410000 | 0.100 | _ | F_ST_ | G4vsG1; G4vsG2 | 11 | *WNT10B, ARF3, TRNAN-GUU, FKBP11, CCDC65, RND1, DDX23, CACNB3, ADCY6, CCNT1, KANSL2* |
|  | 4 | 3 | 116970001 | 117160000 | 0.190 | XP-EHH | _ | G4vsG1; G4vsG2 | 3 | *LRRC10, CCT2, FRS2* |
|  | 5 | 3 | 137060001 | 137330000 | 0.270 | _ | F_ST_ | G4vsG1; G4vsG2 | 1 | *LOC105607476* |
|  | 6 | 3 | 137230001 | 137350000 | 0.120 | _ | F_ST_ | G4vsG1; G4vsG2 | 2 | *CCNT1, KANSL2* |
|  | 7 | 3 | 149900001 | 150090000 | 0.190 | _ | F_ST_ | G4vsG1; G4vsG3 | 1 | *ACSS3* |
|  | 8 | 17 | 18300001 | 18730000 | 0.430 | XP-EHH | _ | G4vsG1; G4vsG2 | 2 | *LOC105602981, LOC105602766* |
|  | 9 | 17 | 18480001 | 18670000 | 0.190 | XP-EHH | _ | G4vsG1; G4vsG2 | 1 | *PCDH18* |
|  | 10 | 17 | 18990001 | 20140000 | 1.150 | XP-EHH | _ | G4vsG1; G4vsG2 | 1 | *LOC105602772* |
|  | 11 | 17 | 19820001 | 20120000 | 0.300 | XP-EHH | _ | G4vsG1; G4vsG2 | 2 | *LOC105602765, LOC105602766* |
|  | 12 | 17 | 25430001 | 25660000 | 0.230 | XP-EHH | _ | G4vsG1; G4vsG2 | 2 | *SLC7A11, PCDH18* |
|  | 13 | 17 | 25560001 | 25810000 | 0.250 | XP-EHH | _ | G4vsG1; G4vsG2 | 1 | *LOC101118305* |
|  | 14 | 18 | 5280001 | 5440000 | 0.160 | _ | F_ST_ | G4vsG1; G4vsG2 | 2 | *CERS3, ADAMTS17* |
|  | 15 | 24 | 27110001 | 27270000 | 0.160 | _ | F_ST_ | G4vsG1; G4vsG3 | 11 | *STX1B, STX4, ZNF668, ZNF646, PRSS53, VKORC1, BCKDK, KAT8, PRSS8, PRSS36, FUS* |
|  | 16 | 27 | 9700001 | 9850000 | 0.150 | _ | F_ST_ | G4vsG2; G4vsG3 | 3 | *AMER1, ASB12, MTMR8* |
|  | 17 | 27 | 44350001 | 44580000 | 0.230 | _ | F_ST_ | G4vsG1; G4vsG2 | 21 | *GRIPAP1, KCND1, OTUD5, PIM2, SLC35A2, PQBP1, TIMM17B, PCSK1N, ERAS, HDAC6, GATA1, GLOD5, TRNAR-CCU, TRNAM-CAU, SUV39H1, WAS, WDR13, RBM3, TBC1D25, EBP, PORCN* |
|  | 18 | 27 | 44390001 | 44580000 | 0.190 | _ | F_ST_ | G4vsG1; G4vsG2 | 2 | *KIAA2022, ABCB7* |
|  | 19 | 27 | 52840001 | 53270000 | 0.430 | _ | F_ST_ | G4vsG1; G4vsG3 | 1 | *FRMPD4* |
|  | 20 | 27 | 53030001 | 53190000 | 0.160 | _ | F_ST_ | G4vsG1; G4vsG3 | 3 | *AMER1, ASB12, MTMR8* |
|  | 21 | 27 | 63460001 | 63690000 | 0.230 | _ | F_ST_ | G4vsG1; G4vsG3 | 4 | *TRNAR-CCU, TRNAM-CAU, SUV39H1, WAS* |
|  | 22 | 27 | 63530001 | 63690000 | 0.160 | _ | F_ST_ | G4vsG1; G4vsG3 | 2 | *KIAA2022, ABCB7* |
|  | 23 | 27 | 92530001 | 92710000 | 0.180 | _ | F_ST_ | G4vsG1; G4vsG3 | 1 | *ZIC3* |
|  | 24 | 27 | 109860001 | 110970000 | 1.110 | _ | F_ST_ | G4vsG1; G4vsG3; G4vsG2 | 5 | *ZCCHC12, IL13RA1, AGTR2, DOCK11, WDR44* |
|  | 25 | 27 | 110930001 | 111080000 | 0.150 | _ | F_ST_ | G4vsG2; G4vsG3 | 1 | *KLHL13* |
|  | 26 | 27 | 128420001 | 128560000 | 0.140 | _ | F_ST_ | G4vsG2; G4vsG3 | 1 | *LOC101102706* |

**Supplementary Table S9** The top most Enriched functional clusters and their enrichment scores following DAVID analysis for group specific genes identified by all methodologies and/or at least two comparisons.

| ***Group*** | ***Category*** | ***ID*** | ***Term*** | ***Gene Count*** | ***P-value*** | ***Genes*** | ***Benjamini*** |
| --- | --- | --- | --- | --- | --- | --- | --- |
| **G1** | KEGG PATHWAY | oas00511 | Other glycan degradation | 1 | 0.0103 | *MAN2C1* | 0.0726 |
|  | KEGG PATHWAY | oas04060 | Cytokine-cytokine receptor interaction | 2 | 0.0110 | *IL13RA1, BMP2* | 0.0726 |
|  | GOTERM_CC_DIRECT | GO:0005737 | cytoplasm | 4 | 0.0464 | *SIN3A, PTPN9, NEIL1, COMMD4* | 0.4071 |
| **G2** | GOTERM_MF_DIRECT | GO:0043565 | sequence-specific DNA binding | 7 | 0.00002 | *HOXA3, HOXA1, EVX1, HOXA7, HOXA6, HOXA5, HOXA4* | 0.00045 |
|  | GOTERM_BP_DIRECT | GO:0009952 | anterior/posterior pattern specification | 5 | 0.00002 | *HOXA10, HOXA9, HOXA3, HOXA2, HOXA6* | 0.00303 |
|  | GOTERM_CC_DIRECT | GO:0005634 | nucleus | 13 | 0.00029 | *KDM1B, DEK, HOXA10, LOC101103215, HOXA9, LATS2, RBL1, HOXA1, CRYM, HOXA7, HOXA6, HOXA5, HOXA4* | 0.00840 |
|  | KEGG PATHWAY | oas05205 | Proteoglycans in cancer | 3 | 0.00317 | *CDC42, HSPG2, WNT4* | 0.14122 |
|  | GOTERM_BP_DIRECT | GO:0006351 | transcription, DNA-templated | 5 | 0.00334 | *LOC101103215, HOXA9, HOXA7, HOXA5, HOXA4* | 0.39266 |
|  | KEGG PATHWAY | oas05100 | Bacterial invasion of epithelial cells | 2 | 0.00514 | *CD2AP, CDC42* | 0.14122 |
|  | GOTERM_BP_DIRECT | GO:0008584 | male gonad development | 3 | 0.00531 | *HOXA10, HOXA9, WNT4* | 0.54771 |
|  | GOTERM_BP_DIRECT | GO:0006355 | regulation of transcription, DNA-templated | 5 | 0.00699 | *HOXA9, HOXA1, HOXA7, HOXA5, HOXA4* | 0.64852 |
| **G3** | KEGG PATHWAY | oas00100 | Steroid biosynthesis | 1 | 0.01879 | *NSDHL* | 0.06847 |
|  | KEGG PATHWAY | oas00534 | Glycosaminoglycan biosynthesis - heparan sulfate / heparin | 1 | 0.01956 | *HS6ST2* | 0.06847 |
|  | GOTERM_CC_DIRECT | GO:0005739 | mitochondrion | 4 | 0.05324 | *DIAPH2, ANXA10, ATP5O, MRPS6* | 0.78386 |
| **G4** | KEGG PATHWAY | oas04130 | SNARE interactions in vesicular transport | 2 | 0.00296 | *STX1B, STX4* | 0.16768 |
|  | KEGG PATHWAY | oas04261 | Adrenergic signaling in cardiomyocytes | 3 | 0.00489 | *CACNB3, AGTR2, ADCY6* | 0.16768 |
|  | KEGG PATHWAY | oas04962 | Vasopressin-regulated water reabsorption | 2 | 0.00513 | *ADCY6, STX4* | 0.16768 |
|  | KEGG PATHWAY | oas05414 | Dilated cardiomyopathy (DCM) | 2 | 0.01901 | *CACNB3, ADCY6* | 0.32947 |
|  | GOTERM_BP_DIRECT | GO:0042312 | regulation of vasodilation | 2 | 0.02121 | *AGTR2, ADCY6* | 1.00000 |
|  | GOTERM_CC_DIRECT | GO:0005622 | intracellular | 7 | 0.02312 | *ARF3, STX1B, DOCK11, AGTR2, ADCY6, ERAS, RND1* | 1.00000 |
|  | KEGG PATHWAY | oas05200 | Pathways in cancer | 4 | 0.03271 | *IL13RA1, ADCY6, PIM2, WNT10B* | 0.32947 |
